# A comparative analysis of complete plastid genomes from *Prangos fedtschenkoi* and *Prangos lipskyi *(Apiaceae)

**DOI:** 10.1002/ece3.4753

**Published:** 2018-12-26

**Authors:** Feruza U. Mustafina, Dong‐Keun Yi, Kyung Choi, Chang Ho Shin, Komiljon Sh. Tojibaev, Stephen R. Downie

**Affiliations:** ^1^ Division of Forest Biodiversity and Herbarium Korea National Arboretum Pocheon Republic of Korea; ^2^ Institute of Botany Uzbek Academy of Sciences Tashkent Republic of Uzbekistan; ^3^ Department of Plant Biology University of Illinois at Urbana‐Champaign Urbana Illinois 61801, USA.

**Keywords:** Apiaceae, chloroplast genome, mitochondrial DNA, plastid DNA, Umbelliferae

## Abstract

*Prangos fedtschenkoi *(Regel & Schmalh.) Korovin and *P. lipskyi* Korovin (Apiaceae) are rare plant species endemic to mountainous regions of Middle Asia. Both are edificators of biotic communities and valuable resource plants. The results of recent phylogenetic analyses place them in *Prangos* subgen. *Koelzella* (M. Hiroe) Lyskov & Pimenov and suggest they may possibly represent sister species. To aid in development of molecular markers useful for intraspecific phylogeographic and population‐level genetic studies of these ecologically and economically important plants, we determined their complete plastid genome sequences and compared the results obtained to several previously published plastomes of Apiaceae. The plastomes of *P. fedtschenkoi* and *P. lipskyi* are typical of Apiaceae and most other higher plant plastid DNAs in their sizes (153,626 and 154,143 bp, respectively), structural organization, gene arrangement, and gene content (with 113 unique genes). A total of 49 and 48 short sequence repeat (SSR) loci of 10 bp or longer were detected in *P. fedtschenkoi* and *P. lipskyi* plastomes, respectively, representing 42–43 mononucleotides and 6 AT dinucleotides. Seven tandem repeats of 30 bp or longer with a sequence identity ≥90% were identified in each plastome. Further comparisons revealed 319 polymorphic sites between the plastomes (IR, 21; LSC, 234; SSC, 64), representing 43.8% transitions (Ts), 56.1% transversions (Tv), and a Ts/Tv ratio of 0.78. Within genic regions, two indel events were observed in *rpo*A (6 and 51 bp) and *ycf*1 (3 and 12 bp), and one in *ndh*F (6 bp). The most variable intergenic spacer region was that of *acc*D/*psa*I, with 21.1% nucleotide divergence. Each *Prangos* species possessed one of two separate inversions (either 5 bp in *ndh*B intron or 9 bp in *pet*B intron), and these were predicted to form hairpin structures with flanking repeat sequences of 18 and 19 bp, respectively. Both species have also incorporated novel DNA in the LSC region adjacent to the LSC/IRa junction, and BLAST searches revealed it had a 100 bp match (86% sequence identity) to noncoding mitochondrial DNA. *Prangos*‐specific primers were developed for the variable *acc*D/*psa*I intergenic spacer and preliminary PCR‐surveys suggest that this region will be useful for future phylogeographic and population‐level studies.

## 1 INTRODUCTION

The genus *Prangos* Lindl. (Apiaceae, Umbelliferae) comprises some 45 species mostly endemic to Asia (Lyskov, Degtjareva, Samigullin, & Pimenov, 2017). They are typically herbaceous, xerophytic plants that perform important roles as edificators of biotic communities. They are also of great economic importance, as many of its members are sources of phytocoumarins, ornamental plants, and fodder for cattle. The plants are diverse morphologically and in their fruit anatomy, resulting in ever‐changing species delimitations and infrageneric classifications (Lyskov et al., 2017).

**Figure 1:**
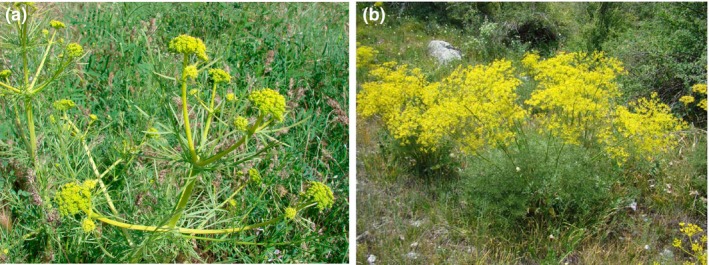
**A.** *Prangos fedtschenkoi*. Piedmont of the Nuratau range (Pamir‐Alay mountain system), Forish district, Jizzakh region, Uzbekistan. Photo by N. Beshko (04 June 2009). **B.** *Prangos lipskyi*. Chatkal range (Western Tien Shan mountain system), Sary ‐ Chelek Nature Reserve, Jalal ‐ Abad Province, Kyrgyzstan. Photo by G. Lazkov (10 July 2015).

*Prangos fedtschenkoi *(Regel & Schmalh.) Korovin and *P. lipskyi *Korovin are rare species endemic to the mountainous regions of Middle Asia (Shishkin, 1950). *P. fedtschenkoi* is more common in the Pamir‐Alay than the Tien Shan mountain systems, with several populations occurring in Kyrgyzstan and Tajikistan (Figure 1a). *P. lipskyi *is an endemic to a narrow geographic area in the Western Tien Shan Mountains and grows on talus slopes (Figure 1b). A recent revision of the genus based on morphological, carpological, and molecular evidence revealed that *P. fedtschenkoi* and *P. lipskyi*, along with three other species, unite as monophyletic in *Prangos* subgen. *Koelzella* (M. Hiroe) Lyskov & Pimenov (Lyskov et al., 2017). In the Bayesian inference tree of molecular data, these two species form two branches of a trichotomy (with the clade of *P. pabularia* Lindl. + *P. ornata* Kuzjmina), suggesting that they may comprise monophyletic sister taxa in other trees. Other molecular systematic studies including *Prangos* species exist (e.g., Banasiak et al., 2013), however, none included representation of *P. fedtschenkoi* or *P. lipskyi*.

Significant declines of both *P. fedtschenkoi *and* P. lipskyi *populations have resulted from human activities, such as agriculture, construction, overgrazing, and collection for pharmaceutical and cosmetological purposes (CACILM, 2006; Limin & Zhang, 2012). *P. fedtschenkoi *is also intensively collected from the wild, as there are currently no plantations of this valuable resource plant. Only limited information is available on the nature of genetic variability in *P. fedtschenkoi* and *P. lipskyi* that can be used to inform conservation strategies, management, and species restoration. Recently, six populations of *P. fedtschenkoi *from Uzbekistan were studied using 10 intersimple sequence repeat (ISSR) markers (Mustafina et al., 2017). A high level of genetic differentiation among populations was detected based on Nei's genetic diversity analysis and AMOVA that was likely caused by range fragmentation as a result of anthropogenic impacts. These factors, along with strong inbreeding, may have decreased within‐population genetic diversity because of low gene flow.

To aid in the development of molecular markers useful for intraspecific phylogeographic and population‐level genetic studies of these rare, but ecologically and economically important plant species, we determined their complete plastid genome sequences, performed a comparative analysis of these data, and compared the results obtained to several previously published plastomes of other Apiaceae species. While the plastomes are typical of Apiaceae and most other eudicot plastid DNAs in their structural organization, gene arrangement, and gene content, variable regions were revealed that will be useful as molecular markers in future studies.

## 2 MATERIALS AND METHODS

### 2.1 Plants materials and DNA extraction

Voucher specimens of field‐collected individuals are as follows: *Prangos fedtschenkoi*, Baysun region, Surkhandarya province, Uzbekistan, 28 June 2015, *Turginov *2310 (TASH) and *Prangos lipskyi*, Chatkal chain, Sari‐Chelek Nature Reserve, Kyrgyzstan, 11 July 2015, *Lazkov *266 (FRU). DNA extractions were conducted using the DNeasy Plant Mini Kit (Qiagen, Hilden, Germany) and checked by electrophoresis in a 1.3% agarose gel, stained with ethidium bromide, and run with a DNA ladder (Thermo Scientific, Waltham, MA, USA). Concentration and quality of DNA were checked using a NanoDrop 2000 spectrophotometer (Thermo Scientific).

### 2.2 Sequencing and plastid genome assembly

Plastid genome sequences were obtained using the Illumina HiSeq 2000 sequencing system and standard protocols. A total of 12,484,132 reads of *P. fedtschenkoi *and 10,849,852 reads of *P. lipskyi *were analyzed. The filtered sequences were assembled using Bowtie 2 v. 2.2.3 (Langmead & Salzberg, 2012) with *Daucus carota* subsp. *sativus* (domesticated carrot; GenBank accession number DQ898156) used as the reference sequence since its plastome has ancestral IR boundaries and no gene rearrangements (Ruhlman et al., 2006). A total of 187,581 reads of *P. fedtschenkoi* and 389,753 reads of *P. lipskyi *were mapped to the reference sequence, with an average coverage of 599.5× for *P. fedtschenkoi *and 1,086× for *P. lipskyi*.

### 2.3 Chloroplast gene annotation

Gene annotations were performed using BLAST (BLASTN, PHI‐BLAST, BLASTX) and the program Dual Organellar Genome Annotator (DOGMA; Wyman, Jansen, & Boore, 2004). Gene nomenclature followed the Chloroplast Genome Database (http://chloroplast.cbio.psu.edu; Cui et al., 2006). Complete plastid genomes have been deposited in GenBank (*P. fedtschenkoi*, KY652265; *P. lipskyi*, KY652266) using Sequin v. 15.50 (6 January 2017). Circular genome maps were drawn using OrganellarGenomeDRAW (Lohse, Drechsel, Kahlau, & Bock, 2013), with nomenclature and arrangement of the plastome's major structural components following convention. Sequences of six Apiaceae plastomes, that is, *D. carota*, *Petroselinum crispum *(HM596073), *Coriandrum sativum *(KR002656), *Prangos trifida *(NC037852), and the two newly sequenced plastomes of *P. fedtschenkoi *and *P. lipskyi*, were compared to align IR single‐copy boundary positions.

### 2.4 Sequence analysis

AT content and codon usage were obtained using MEGA6 v. 6.06 (Tamura, Stecher, Peterson, Filipski, & Kumar, 2013). Plastid genomes were aligned using Mauve v. 2.3.1 (Darling, Mau, & Perna, 2010) and sequences of the protein‐coding genes and intergenic spacer (IGS) regions were extracted. Nucleotide diversity and Ka/Ks values of protein‐coding regions were analyzed using DnaSP v. 5.10.01 (Librado & Rozas, 2009) and MEGA6. Polymorphic sites (Ts and Tv) were analyzed by pairwise comparison of each protein‐coding region, intergenic spacer, and intron using MUSCLE (Edgar, 2004) and the SNP finder program integrated into Geneious v. 10.3 (Kearse et al., 2012). To determine the origin of the novel plastid DNA fragments adjacent to IRa/LSC, these sequences were checked against the NCBI nucleotide DNA database using BLAST.

### 2.5 *Prangos*‐specific primers

Primers specific for the *acc*D/*psa*I IGS region in *Prangos* were developed with software Primer3 v. 2.3.7 (Untergasser et al., 2012) and synthesized by Macrogen, Korea. Three pairs of primers located in the spacer region between *acc*D and *psa*I were considered, with the goal of identifying those primer pairs yielding the best results. PCR was conducted with the following regime: initial denaturation at 94°C (1 min), followed by 35 cycles of denaturation at 94°C (30 s), annealing at 54°C (40 s), extension at 72°C (40 s), and a final extension at 72°C (45 min). PCR products of the *acc*D/*psa*I intergenic spacer region were obtained for 46 additional accessions belonging to 16 Middle Asian *Prangos *species, including those from geographically remote populations. *P. fedtschenkoi *was represented by 10 accessions (from Kyrgyzstan, Tajikistan, and Uzbekistan) and *P. lipskyi *was represented by three accessions (from Kyrgyzstan and Uzbekistan).

### 2.6 Repeat and inversion analyses

Short sequence repeats (SSRs) were analyzed with Phobos v. 3.3.12 (Mayer, Leese, & Tollrian, 2010) using the default mismatch and a gap score of −5, with a size threshold ≥10 bp. Repeating sequences were analyzed by REPuter (Kurtz et al., 2001) and Tandem Repeats Finder v. 4.07b (Benson, 1999). Direct and inverted (palindromic) repeats were identified with a size ≥30 bp and a Hamming distance of 3 (limiting hits to sequence identity of ≥90%). Secondary structure was predicted by mFOLD (Zuker, 2003). To survey for the presence of identical tandem repeats and the two small inversions and their associated flanking repeat regions identified in the plastomes of *P. fedtschenkoi* and *P. lipsky* in related taxa, the complete plastid genomes of 23 other Apiaceae species (represented by 25 accessions) and two Araliaceae species were downloaded from NCBI's public database. Sequence similarity searches and alignments were carried out using Clustal W (Thompson, Higgins, & Gibson, 1994). Repeating sequences were analyzed by REPuter, and the forms and stabilities of hairpin structures were evaluated by MFOLD.

## 3 RESULTS

### 3.1 General plastome features

**Figure 2:**
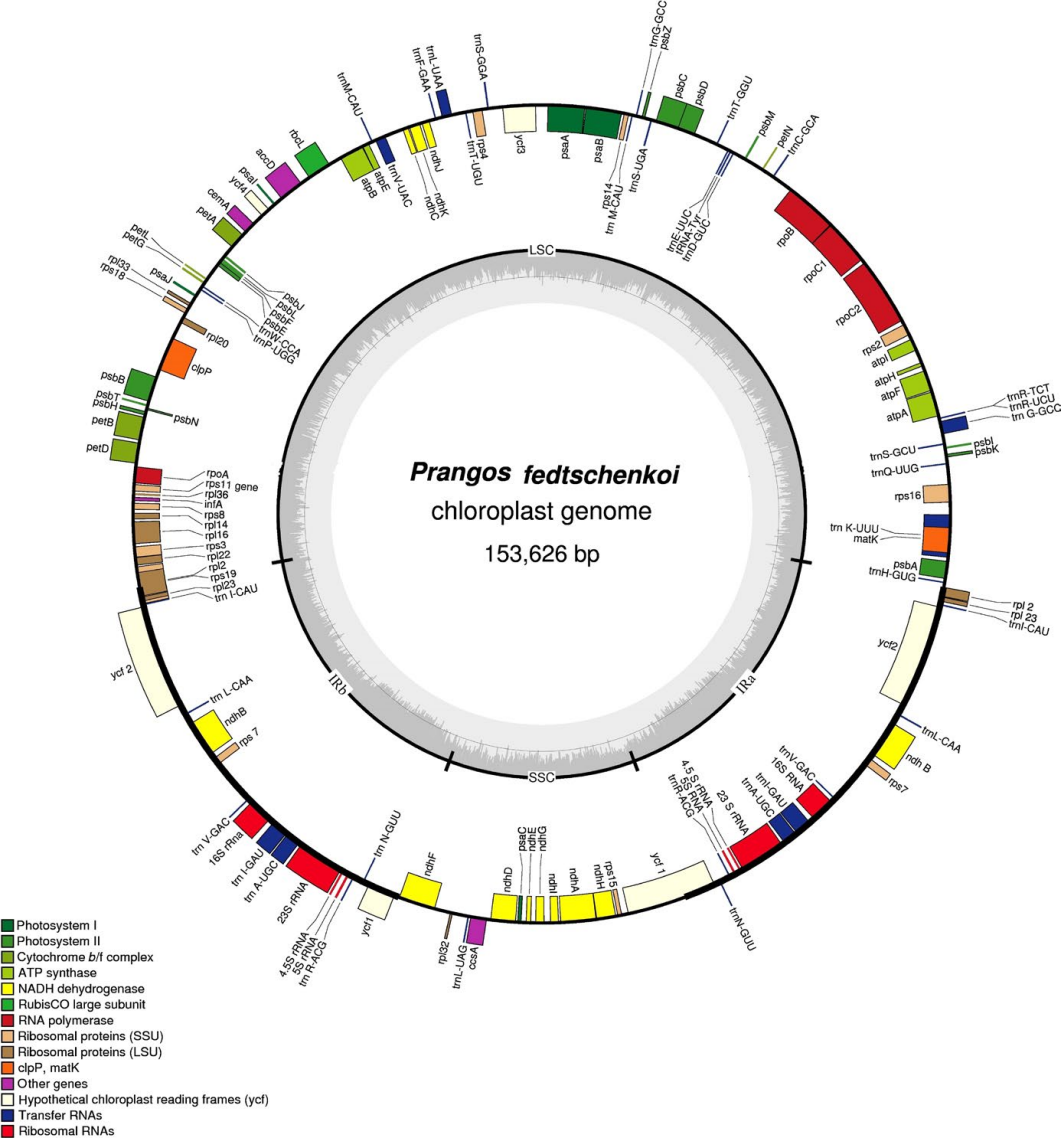
Circular plastome map of *Prangos fedtschenkoi*. Genes are classified into 14 groups according to their biological function and are shown by different colored boxes. Genes transcribed clockwise are shown inside of the circle; genes transcribed counter‐clockwise are shown outside of the circle. The small single‐copy (SSC) region is 17,494 bp in size, the large single‐copy (LSC) region is 85,614 bp in size, and each inverted repeat (IRa, IRb) region is 25,259 bp in size. The internal gray circle indicates GC content and the thin circular line marks the 50% threshold. The nucleotide sequence of the *P. fedtschenkoi* chloroplast genome appears under the accession number KY652265 in the DDBJ/GenBank databases

**Figure 3:**
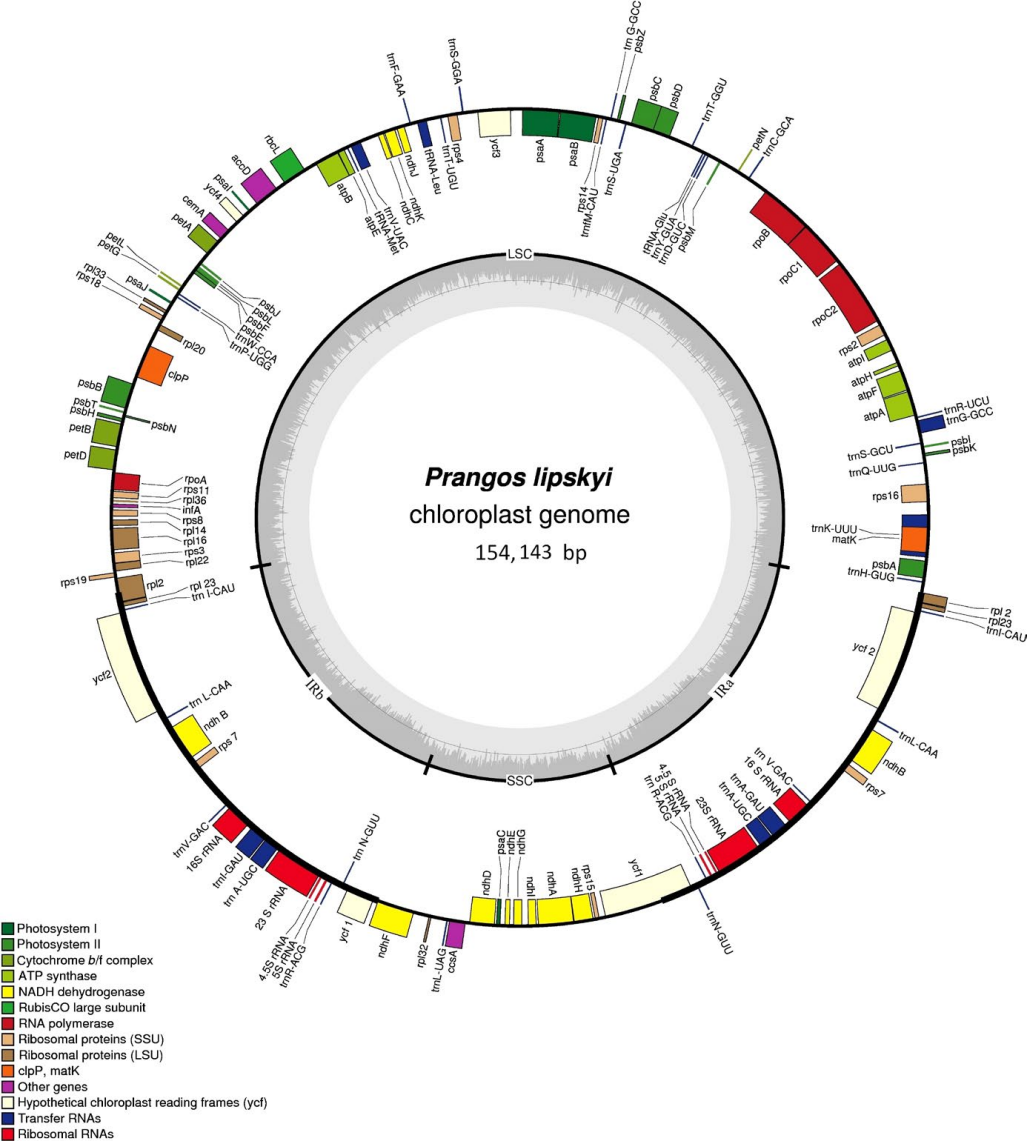
Circular plastome map of *Prangos lipskyi*. Genes are classified into 14 groups according to their biological function and are shown by different colored boxes. Genes transcribed clockwise are shown inside of the circle; genes transcribed counter‐clockwise are shown outside of the circle. The small single‐copy (SSC) region is 17,402 bp in size, the large single‐copy (LSC) region is 86,131 bp in size, and each inverted repeat (IRa, IRb) region is 25,305 bp in size. The internal gray circle indicates GC content and the thin circular line marks the 50% threshold. The nucleotide sequence of the *P. lipskyi* chloroplast genome appears under the accession number KY652266 in the DDBJ/GenBank databases

The plastomes of *P. fedtschenkoi *and *P. lipskyi* are typical of most other nonmonocot angiosperm plastid DNAs in their structural organization, gene arrangement, and gene content (Figures 2 and 3). They have two large inverted repeats (IRs), separated by large single‐copy (LSC) and small single‐copy (SSC) regions. A comparison of their major structural features, to each other and to *P. trifida* and *D. carota,* is presented in Table 1. The plastomes of *P. fedtschenkoi *and *P. lipskyi* range in size from 153,626 bp in *P. fedtschenkoi* to 154,143 bp in *P. lipskyi*, with the latter having a substantially larger LSC region. Both plastomes share identical compliments of genes, each with four unique rRNA genes, 30 unique tRNA genes, and 79 unique protein‐coding genes. A list of unique genes present in both plastomes, as represented by *P. fedtschenkoi*, is presented in Table 2. Allowing for duplication of genes in the IR and open reading frames (ORFs), each plastome contains 134 complete, predicted coding regions. Comparing the *Prangos* plastome structural features to those of *D. carota* reveals major differences in sizes of all major structural components and total number of predicted coding regions (Table 1).

**Table 1:** Comparison of major structural features of the plastomes of *Prangos fedtschenkoi*, *Prangos lipskyi, Prangos trifida, *and *Daucus carota*

Feature	*P. fedtschenkoi*	*P. lipskyi*	*P. trifida*	*D. carota*
Entire plastome size (bp)	153,626	154,143	153,510	155,911
IR size (bp)	25,259	25,305	24,792	27,051
LSC region size (bp)	85,614	86,131	86,481	84,242
SSC region size (bp)	17,494	17,402	17,445	17,567
Number of coding regions	134	134	134	136
Number of genes	113	113	113	115
Number of protein‐coding genes	79	79	79	81
Number of genes duplicated in the IR	21	21	21	21
Number of pseudogenes	2 (*rpl*2, *ycf*1)	2 (*rpl*2, *ycf*1)	2 (*rpl*2, *ycf*1)	2 (*rps*19, *ycf*1)
Number of open reading frames	4	4	4	4
Number of *tRNA* genes	30	30	30	30
Number of *rRNA *genes	4	4	4	4
Number of genes with intron(s)	18	18	18	18

**Table 2:** Unique genes of the *Prangos fedtschenkoi* plastome

Group of gene	Name of gene	Number of genes
RNA genes		
Ribosomal RNAs	*rrn*4.5 (x2), *rrn*5 (x2), *rrn*16 (x2), *rrn*23 (x2)	4
Transfer RNAs	*trn*A‐UGC(x2)^a^, *trn*C‐GCA, *trn*D‐GUC, *trn*E‐UUC, *trn*F‐GAA, *trn*fM‐CAU, *trn*G‐GCC, *trn*G‐GCC^a^, *trn*H‐GUG, *trn*I‐CAU (x2), *trn*I‐GAU (x2)^a^, *trn*K‐UUU^a^, *trn*L‐CAA (x2), *trn*L‐UAA^a^, *trn*L‐UAG, *trn*M‐CAU, *trn*N‐GUU (x2), *trn*P‐UGG, *trn*Q‐UUG, *trn*R‐ACG (x2), *trn*R‐UCU, *trn*S‐GCU, *trn*S‐GGA, *trn*S‐UGA, *trn*T‐GGU, *trn*T‐UGU, *trn*V‐GAC(x2), *trn*V‐UAC^a^, *trn*W‐CCA, *trn*Y‐GUA	30
Protein genes		
Photosynthesis		
Photosystem I	*psa*A, *psa*B, *psa*C, *psa*I, *psa*J, *ycf*3^b^	5
Photosystem II	*psb*A, *psb*B, *psb*C, *psb*D, *psb*E, *psb*F, *psb*H, *psb*I, *psb*J, *psb*K, *psb*L, *psb*M, *psb*N, *psb*T, *psb*Z	15
Cytochrome b/f complex	*pet*A, *pet*B^a^, *pet*D^a^, *pet*G, *pet*L, *pet*N	6
NADH‐dehydrogenase	*ndh*A^a^, *ndh*B(x2)^a^, *ndh*C, *ndh*D, *ndh*E, *ndh*F, *ndh*G, *ndh*H, *ndh*I, *ndh*J, *ndh*K	11
ATP synthase	*atp*A, *atp*B, *atp*E, *atp*F^a^, *atp*H, *atp*I	6
Large subunit of Rubisco	*rbc*L	1
ATP‐dependent protease	*clp*P^b^	1
Envelope membrane protein	*cem*A	1
Ribosomal proteins		
Large units	*rpl*2 (x2, part)^a^, *rpl*14, *rpl*16^a^, *rpl*20, *rpl*22, *rpl*23 (x2), *rpl*32, *rpl*33, *rpl*36	9
Small units	*rps*2, *rps*3, *rps*4, *rps*7 (x2), *rps*8, *rps*11, *rps*12^a^ (x2), *rps*14, *rps*15, *rps*16^a^, *rps*18, *rps*19	12
Transcription/translation		
DNA‐dependent RNA polymerase	*rpo*A, *rpo*B, *rpo*C1^a^, *rpo*C2	4
Miscellaneous proteins	*acc*D, *ccs*A, *inf*A, *mat*K	4/75
Hypothetical proteins and Conserved reading frame	*ycf*1 (x2, part), *ycf*2 (x2), *ycf*4	3/4
Total number of unique genes		113

a Genes containing one intron.

b Genes containing two introns.

In *P. fedtschenkoi*, 57.62% of the plastome consists of gene‐coding regions (54.02% proteins and 3.6% RNAs). In *P. lipskyi*, 57.61% of the plastome is gene‐coding (54.01% proteins and 3.6% RNAs). Noncoding regions comprise approximately 42.4% of each plastome (11.3% intron and 31.1% IGS regions). The overall AT content in each *Prangos* species was 62.3% (Table 3). The AT content of the IR (57.1%) is lower than that of both LSC (64.0%) and SSC (68.7%) regions. Within protein‐coding regions of *P. fedtschenkoi*, the AT content is higher at the third codon position (63.0%), whereas in *P. lipskyi *the AT content is lowest at the third codon position (61.7%). The codon usage pattern suggests that codons containing A/T in the third position are given preference in *P. fedtschenkoi *and *P. lipskyi* (Supporting Information Tables S1 and S2), in accordance with the codon usage pattern of the majority of eudicot species.

**Table 3:** Comparison of nucleotide composition between *Prangos fedtschenkoi* and *Prangos lipskyi* plastomes

	Nucleotide composition of whole genome (%)						AT content (%)			AT content in codon regions (%)		
	T	C	A	G	AT	GC	LSC	IR	SSC	1	2	3
*P. fedtschenkoi*	31.4	19.2	30.8	18.5	62.3	37.7	64.0	57.1	68.7	61.6	61.9	63.0
*P. lipskyi*	31.4	19.2	30.8	18.5	62.3	37.7	64.0	57.1	68.7	62.9	62.9	61.7

### 3.2 IR single‐copy boundary positions

**Figure 4:**
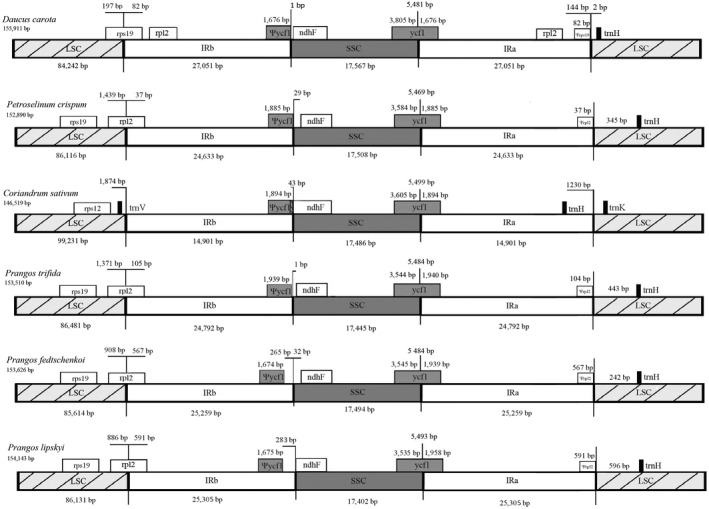
Schematic comparison of the large single‐copy (LSC), inverted repeat (IRa, IRb), and small single‐copy (SSC) regions among six Apiaceae plastid genomes: *Daucus carota*, *Petroselinum crispum*, *Coriandrum sativum*, *Prangos trifida*, *Prangos fedtschenkoi*, and *Prangos lipskyi.* Genes overlapping or flanking IR single‐copy junctions are identified (their sizes do not correlate with their actual length). Pseudogenes are denoted by Ψ (i.e., Ψycf1, Ψrps19, Ψrpl2)

A comparison of IR single‐copy boundary positions of the three *Prangos* species, *D. carota*, and two additional species of the family Apiaceae (*Petroselinum crispum*, parsley; *Coriandrum sativum*, coriander) is presented in Figure 4. In *Prangos* spp., the IRb/LSC boundary falls in gene *rpl*2, which results in *rpl*2 pseudogenes of 567 bp in *P. fedtschenkoi* and 591 bp in *P. lipskyi* in IRa adjacent to the IRa/LSC junction. These *rpl*2 pseudogenes have lost the second exon of the gene. A similar IRb/LSC boundary position in *rpl*2 occurs in *P. trifida *and *P. crispum*. *D. carota *has the ancestral IRb/LSC boundary position in or near *rps*19. A considerable contraction of the IR occurs in *C. sativum*. Here, IRa/LSC is located downstream of *trn*K‐UUU, the positions of *trn*H‐GUG and *psb*A are contained within the IR, and the IRb/LSC border falls downstream of *rps*12 and *trn*V‐GAC. In all species, the IRa/SSC boundary position occurs in *ycf*1, creating *ycf*1 pseudogenes ranging between 1,674 bp (*P. fedtschenkoi*) and 1,939 bp (*P. trifida*) in IRb at the IRb/SSC boundary position.

### 3.3 Plastid genome sequence divergence

The average sequence divergence between entire plastomes of *P. fedtschenkoi *and *P. lipskyi *was 0.6%. Average sequence divergences between *P. fedtschenkoi *and *D. carota* and between *P. lipskyi *and *D. carota* were 9.1% and 9.9%, respectively. The average divergence values of the IR regions between *P. fedtschenkoi *and *P. lipskyi*, between *P. fedtschenkoi *and *D. carota*, and between *P. lipskyi *and *D. carota *were 0.1%, 9.0%, and 9.5%, respectively, while divergence values of the LSC regions were 1.1%, 9.1%, and 11.4%, respectively, and divergence values of the SSC regions were 1.1%, 9.4%, and 9.3%, respectively.

### 3.4 Coding region divergence

Detailed sequence comparisons of 83 protein‐coding genes between *P. fedtschenkoi *and *P. lipskyi *are provided in Supporting Information Table S3. Average Ka/Ks ratios for these gene regions were 0.3988 across entire plastomes, 0.2857 for the IRs, and 0.2476 for the LSC and 0.6795 for the SSC regions. Sequence divergence values of each protein‐coding gene region between *P. fedtschenkoi *and *P. lipskyi *varied from identity to 1.3% (reaching 0.1% in the IR, 1.3% in the LSC, and 0.7% in the SSC). Genes showing highest sequence divergence included *rpo*A (1.3%), *psb*T (1.0%), *psb*I (0.9%), and *ycf*1 (0.7%). Genes *rpo*A, *ycf*1, and *ndh*F exhibited length difference in pairwise comparisons resulted from multiple indels and length variation (of 57, 15, and 6 bp, respectively).

### 3.5 Intergenic spacer region divergence

*Prangos *plastomes contain 108 intergenic spacer regions longer than 10 bp in length (Supporting Information Table S4). Sequence divergence values of these IGS regions ranged from identity to 21.1% (reaching 6.9% in the IR, 21.1% in the LSC, and 19.9% in the SSC). The most divergent IGS regions are *acc*D/*psa*I (21.1%), *psb*Z/*trn*G‐GCC (13.2%), *rps*16/*trn*Q‐UUG (8.5%), and *rps*14/*psa*B (7.3%) in the LSC, *rps*19/*rpl*2 (6.9%) in the IR, and *ndh*E/*ndh*G (19.9%) in the SSC.

Small‐ and medium‐sized insertion and deletion events prevailed over long length indels in IGS regions between the *P. fedtschenkoi* and *P. lipskyi* plastomes*. *The total length of all indel events varied from 7 bp in the IR, 119 bp in the SSC, to 641 bp in the LSC, totaling 767 bp. Medium‐sized indel events (20–40 bp) occurred in *psb*Z/*trn*G‐GCC (38 bp), *acc*D/*psa*I (34 bp), and *pet*A/*psb*J (23 bp) in the LSC, and in *ndh*F/*rpl*32 (20 bp) in the SSC. Longer indel events were observed in *rps*16/*trn*Q‐UUG (69 bp) and *acc*D/*psa*I (57 and 11 bp) in the LSC, and in *ndh*E/*ndh*G (50 bp) in the SSC.

### 3.6 Intron region divergence

Detailed sequence comparisons among intron regions in *P. fedtschenkoi *and *P. lipskyi *are provided in Supporting Information Table S5. The most conserved intron, showing no variation, is that within 3’*rps*12 of the IR. Intron diversity levels varied from identity to 7.4% (from identity to 0.9% in the IR, from 0.3% to 7.4% in the LSC, and was 0.4% [*ndh*A] in the SSC). The most divergent intron regions were those in genes *trn*L‐UAA (7.4%), *rpl*16 (2.7%), *ycf*3 (2.3%), and *clp*P (2.3%) in the LSC and *ndh*B (0.9%) in the IR. The length of all intron indel events totaled 118 bp. The majority of indels were less than 20 bp. One longer sized indel was located in the *trn*L‐UAA intron (32 bp).

### 3.7 Polymorphic sites

Data on polymorphic sites within the two *Prangos* plastomes are presented in Supporting Information Tables S3, S4 and S5. The total number of polymorphic sites in the protein‐coding regions was 121, including seven in the IR, 68 in the LSC, and 46 in the SSC. Of these, 51.2% are Ts and 48.8% are Tv. The total number of the polymorphic sites in the intergenic spacer regions was 145, including five in IR, 124 in LSC, and 16 in SSC. The total number of the polymorphic sites in all introns is 53, including nine in the IR, 42 in the LSC, and two in the SSC. Overall, the total number of polymorphic sites in all regions of the plastome is 319, including 21 in the IR, 234 in LSC, and 64 in SSC (representing 43.8% Ts and 56.1% Tv).

### 3.8 Development of *Prangos*‐specific primers

In pairwise comparisons of sequences *P. fedtschenkoi* and *P. lipskyi*, the *acc*D*/psa*I IGS region was deemed most variable, with a level of nucleotide divergence of 21.1%. This region also includes five indel events, of 6, 11, 11, 34, and 57 bp (Supporting Information Table S4). With these high levels of nucleotide and length variation, the region was considered further for future use in low‐level taxonomic and population studies. Of the three pairs of *Prangos*‐specific primers considered (Table 4), the *acc*D/*psa*I_3F and *acc*D/*psa*I_3R primer pair produced the strongest, single product in PCR amplifications of 46 additional accessions belonging to 16 *Prangos* species from Middle Asia, including those from geographically remote populations (Mustafina et al., unpublished data). Comparisons of protein‐coding genes, intergenic spacer regions, and introns among *P. fedtschenkoi, P. lipskyi*, and *P. trifida *plastomes provided evidence of high sequence variability within *acc*D*/psa*I (16.5%), *rps*16/*trn*Q‐UUG (16.5%), and *psb*Z/*trn*G‐GCC (11.4%) IGS regions (Supporting Information Tables S6, S7 and S8). In the *P. trifida *plastome, the *pet*A/*psb*J IGS region was also highly variable (26.9%); however, the high variability of this region is a result of a unique 315 bp insertion.

**Table 4:** Three pairs of *Prangos*‐specific primers developed for the *acc*D/*psa*I intergenic spacer region for use in future low‐level taxonomic and population studies. For each primer pair, forward (F) and reverse (R) primers are indicated

Primer	Sequence	Binding region	Tan (°C)	GC (%)
*acc*D/*psa*I_1F	5’‐TGGGAGATATCATTATTGCC‐3’	59,651–59,670	54.3	40.0
*acc*D/*psa*I_1R	5’‐ GCAATGGCTTCTTTATTTCT‐3’	60,544–60,563	52.3	35.0
*acc*D/*psa*I_2F	5’‐CGCTTTCTTTCCTTTGAATC‐3’	59,844–59,863	54.3	40.0
*acc*D/*psa*I_2R	5’‐GTAGGCTTAGTATTTCCGG‐3’	60,520–60,538	55.2	47.3
*acc*D/*psa*I_3F	5’‐TTAATCGTACCACGTAATCC‐3’	59,788–59,807	54.3	40.0
*acc*D/*psa*I_3R	5’‐GCAATGGCTTCTTTATTTCT‐3’	60,544–60,563	52.3	35.0

Note

Binding region: accD/*psa*I spacer region within Mauve alignment of *P. fedtschenkoi *and *P. lipskyi *plastomes; Tan: annealing temperature.

### 3.9 Insertions of novel DNA

Unique, noncoding DNA of 242 bp in *P. fedtschenkoi*, 596 bp in *P. lipskyi*, and 443 bp in *P. trifida* occurs between gene *trn*H‐GUG (LSC) and the IRa/LSC junction (Figure 4). BLAST searches of these novel DNA sequences did not show any significant similarity to any other known plastid DNA sequence. Instead, the BLAST results revealed small, significant matches to mitochondrial DNA (specifically, to an IGS region adjacent to mitochondrial gene cytochrome b [*cob*] in *D. carota*). The results of a BLAST search of the 242 bp novel insert in *P. fedtschenkoi* are shown in Table 5. Both *P. fedtschenkoi* and *P. lipskyi* plastomes showed a 100 bp match having 86% sequence similarity to this mtDNA region. The *P. trifida* plastome showed a 102 bp match having 88.2% sequence similarity to this same region. Alignment of these *Prangos* novel DNA fragments showing similarities to *D. carota* mtDNA (i.e., positions 147–242 bp in *P. fedtschenkoi*, 501–596 bp in *P. lipskyi*, and 348–443 bp in *P. trifida*) with a 101 bp fragment of the IGS region adjacent to *D. carota* mitochondrial gene *cob *showed 85 identical sites across the four sequences compared (Supporting Information Figure S1).

**Table 5:** Results of BLAST searches of the nucleotide database (as of 3 October 2018) querying the 242 bp novel insertion sequence in *Prangos fedtschenkoi. *Only hits with lengths ≥100 bp and a percent similarity at least 86% are shown

Accession	Species	Location	Length of match (bp)	Percent similarity
AY007816	*Daucus carota*	cytochrome b *(cob)*; ORF25 (orf25)	100	86
AY007821	*Daucus carota*	ATPase8 *(ATP8)*; cytochrome b *(cob)*	100	86
JQ248574	*Daucus carota *subsp*. sativus*	cytochrome b *(cob)*; ORF25 (orf25)	100	86

### 3.10 Repeat analysis

Repeat analysis identified 49 short motif SSRs in *P. fedtschenkoi *and 48 SSRs in *P. lipskyi *plastomes having repeat lengths ≥10 bp (Table 6). In *P. fedtschenkoi, *43 of these were mononucleotides and six were AT dinucleotides, and in *P. lipskyi* 42 were mononucleotides and six were AT dinucleotides. Repeat lengths of A and T classes of mononucleotides were 10–17 bp and 10–14 bp, respectively. In *P. lipskyi, *a poly‐C nucleotide repeat of 11 bp was found in the *trn*A‐UGC intron (IRa) and a poly‐G nucleotide repeat of 11 bp occurred in the *trn*A‐UGC intron (IRb). AT dinucleotide repeats with lengths of 10–14 bp were found in *P. fedtschenkoi* and *P. lipskyi*, with a repeat number of 6.

**Table 6:** Features of short sequence repeats (SSRs) with repeat length ≥10 bp in *Prangos fedtschenkoi* and *Prangos lipskyi *plastomes. Repeats include both copies of the IR

Unit size	Repeat length, bp	Repeat number	
		*P. fedtschenkoi*	*P. lipskyi*
A	10–17	20	17
T	10–14	23	23
C	11	—	1
G	11	—	1
AT	10–14	6	6

The plastomes of *P. fedtschenkoi *and *P. lipskyi* each had seven large tandem repeats, with repeat size ≥30 bp and sequence identity >90% (Tables 7 and 8). Repeat units are repeated 2–4 times, with the majority occurring in the LSC region. Among the ten uniquely occurring large tandem repeats identified in *Prangos*, most were palindromic dispersed. Four tandem repeats have identical features and are located within the same genomic regions in both plastomes; two of these occur in IGS regions and two others occur in coding sequence regions. A survey for these 10 *Prangos* large tandem repeats in 27 other accessions of Apiaceae and Araliaceae resulted in three (i.e., repeat nos. 4, 5, and 6 in *P. fedtschenkoi* [Table 7] and repeat nos. 3, 5, and 6 in *P. lipskyi* [Table 8]) being widely distributed (Supporting Information Table S9); one of these occurs in the *pet*N/*psb*M IGS region, and the other two occur in coding sequence regions. An additional repeat occurring in both *psb*M/*trn*D‐GUC and *trn*E‐UUC/*trn*T‐GGU IGS regions was shared by *P. fedtschenkoi*, *P. lipskyi*, *P. trifida*, and *Crithmum maritimum. P. fedtschenkoi *and *P. trifida *share a repeat within the *trn*L‐UAA intron.

**Table 7:** Features of large tandem repeat loci (≥30 bp) in the *Prangos fedtschenkoi* plastome

Repeat #	Repeat unit	Repeat number	Size (bp)	Repeat type	Location
1	ATTGACGAGCTACAGCACTCGCACCTATTAACGCAACTAAAAGAATTATT	2	100	Forward	IGS (*ndh*E ‐ *ndh*G)
2	AAAAGGGAAAGATGATGGATGTACTTATTGAATCTGTCG	2	78	Palindromic dispersed	**IGS** (*psb*M – *trn*D‐GUC; *trn*E‐UUC ‐ *trn*T‐GGU)
3	ATACGTATGTATATAC	2	32	Forward	Intron (*trn*L‐UAA)
4	GAGGATATTGATGCTAGTGAGGATATTGATGCTAGTGA	4	76 (2×)	Palindromic dispersed	**CDS** (*ycf*2)
5	ACGGAAAGAGAGGGATTCGAACCCTCGGTA	2	60	Palindromic dispersed	**CDS** (*trn*S‐GCU; *trn*S‐GGA)
6	GTAAGAAAGAAATAT	2	30	Palindromic	**IGS** (*pet*N – *psb*M)
7	ATATTCATAAAGTAATGATA	2	40	Forward	IGS (*ndh*F *– rpl*32)

Notes

The bold characters represent the shared tandem repeats with *P. lipskyi* chloroplast genome*.*

CDS: coding DNA sequence; IGS: intergenic sequence.

**Table 8:** Features of large tandem repeat loci (≥30 bp) in the *Prangos lipskyi *plastome

Repeat #	Repeat unit	Repeat number	Size (bp)	Repeat type	Location
1	ATCATATAAATACAAAGATTATATTCATAATTCTATTCAT	2	40	Palindromic dispersed	IGS (*psb*Z ‐ *trn*G‐GCC; *psa*C – *ndh*E)
2	AAAAGGGAAAGATGATGGATGTACTTATTGAATCTGTCG	2	78	Palindromic dispersed	**IGS** (*psb*M – *trn*D‐GUC; *trn*E‐UUC ‐ *trn*T‐GGU)
3	GAGGATATTGATGCTAGTGAGGATATTGATGCTAGTGA	4	76 (2×)	Palindromic dispersed	**CDS** (*ycf*2)
4	ATGATATATGCTTTTGTACCTTCTATACTCACTTAG	2	72	Forward dispersed	IGS (*acc*D ‐ *psa*I)
5	ACGGAAAGAGAGGGATTCGAACCCTCGGTA	2	30	Palindromic dispersed	**CDS** (*trn*S‐GCU; *trn*S‐GGA)
6	GTAAGAAAGAAATAT	2	30	Palindromic	**IGS** (*pet*N ‐ *psb*M)
7	ATGATAAAAAATGGACATTATGA	2	46	Forward	IGS (*pet*A – *psb*J)

Notes

The bold characters represent the shared tandem repeats with *P. fedtschenkoi* chloroplast genome.

CDS: coding DNA sequence; IGS: intergenic sequence.

### 3.11 Stem–loop hairpin structures

**Figure 5:**
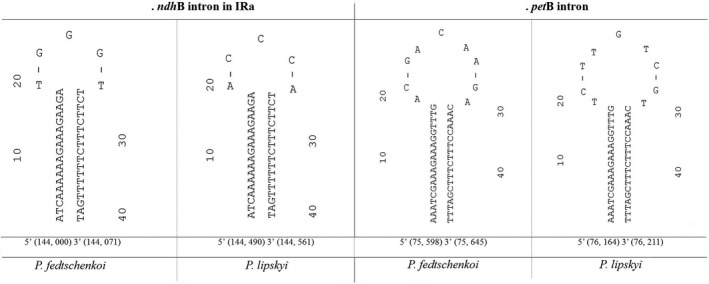
Short inversion mutations and associated secondary structure between *Prangos fedtschenkoi *and *Prangos lipskyi* plastomes. Inversions are located within intron regions of *ndh*B (IRb, IRa) and *pet*B. Free energy of the secondary structures: (a) dG = −16.42 kcal/mol; dG = −16.72 kcal/mol; (b) dG = −19.37 kcal/mol, dG = −19.98 kcal/mol

Each *Prangos* species possessed one of two small inversions, of either 5 or 9 bp (Figure 5). The first occurs in the *P. lipskyi ndh*B intron in the IR, thus there are two copies of this inversion present; each copy has repeat sequences of 18 bp at its ends. The second inversion occurs in the *P. fedtschenko*i *pet*B intron and has repeat sequences of 19 bp at its ends. All regions form distinct stem–loop hairpin structures, with the sequences of the loop regions flip‐flopped in each *Prangos* species.

Results of a survey for the presence of these two small inversions and their associated flanking inverted repeats in other Apiaceae and Araliaceae accessions are presented in Supporting Information Tables S10 and S11. The 5 bp inversion of *P. lipskyi*, represented by sequence ACCCA, is also detected in *P. trifida*, while similar sequences (e.g., ACTCA) are present in *Anthriscus*, *Carum*, *Coriandrum*, and Araliaceae. In contrast, the sequence TGGGT of *P. fedtschenkoi,* or TGAGT with 1 bp change, occurs in 20 other accessions, including *Bupleurum*, the most basal taxon within Apiaceae subfamily Apioideae. The 9 bp inversion of *P. fedtschenkoi*, with sequence ACGACAAGA, also occurs in *Anethum graveolens*, *Carum carvi*, and *Foeniculum vulgare*, and with 1 bp change (ACGAAAAGA) in *Hansenia* spp. and *Tiedemannia filiformis*. In contrast, the loop sequence of *P. lipskyi *and *P. trifida*, TCTTGTCGT, and similar sequence TCTTTTCGT, occurs in multiple other taxa. While the congeners of *Angelica* (4 spp.), *Bupleurum* (2 spp.), and *Hansenia* (4 spp.) are each represented by the same loop sequence for each of the two inversions, these loop regions are actually quite variable, with multiple motifs represented.

## 4 DISCUSSION

The results of this study have multiple, important implications. First, entire plastome sequences were determined for two endemic species of *Prangos* from Middle Asia: *P. fedtschenkoi, *a valuable resource plant; and *P. lipskyi*, a narrow endemic species of the Western Tien Shan Mountains. *Prangos* is treated in the *Cachrys* clade of Apiaceae based on molecular phylogenetic study (Downie, Spalik, Katz‐Downie, & Reduron, 2010). A comparative analysis of these plastomes identified new loci, specifically the *acc*D/*psa*I IGS region, that offer levels of variation higher than those regions commonly employed in molecular phylogenetic studies of the family. Second, the *Prangos*‐specific primers developed and surveyed for the *acc*D/*psa*I IGS region will be useful for future phylogeographic and population‐level studies. Similarly, the unique short repeated regions described for each plastome will be helpful to understand the genetic potential of each population, which is important in the development of ex situ conservation strategies. Third, the incorporation of novel DNA at the LSC/IRa junction suggestive of mtDNA adds to the growing body of evidence showing introgression of mtDNA into the angiosperm plastome. Fourth, the presence of two different inversions in sister species, each in one species but not the other, provides another marker that can be surveyed in a greater sampling of the group. However, similar inversions can occur in parallel in more distantly related taxa, thus reducing their phylogenetic utility.

### 4.1 Identification of variable loci

Direct comparisons of 83 protein‐coding regions, 113 IGS regions, and 18 introns from three complete *Prangos* plastomes resulted in the identification of seven loci having high nucleotide and length variability: *acc*D/*psa*I, *psb*Z/*trn*G‐GCC, *rps*16/*trn*Q‐UUG, *rps*14/*psa*B, *ndh*E/*ndh*G, *pet*A/*psb*J, and the *trn*L‐UAA intron. Shaw et al. (2005) have discussed that the ideal plastid region for phylogenetic investigation should combine high variability with fragments of approximately 700–1,500 bp in size that will permit ease of PCR amplification. Using these criteria, the regions most suitable for phylogenetic inference and population‐level analyses in *Prangos* are *acc*D/*psa*I, *psb*Z/*trn*G*, rps*16/*trn*Q, and the *trn*L intron. Within Apiaceae, the plastid loci most frequently employed in molecular phylogenetic studies to date have included the *rpl*16, *rpo*C1, and *rps*16 introns and the *trn*H/*psb*A, *trn*D/*trn*T, and *trn*F/*trn*L/*trn*T IGS regions (Downie & Jansen, 2015). In *Prangos*, *acc*D/*psa*I, and *psb*Z/*trn*G are more variable and better suited for intraspecific and population‐level studies than these other loci. Indeed, the *acc*D/*psa*I IGS region was listed among the most variable loci identified from the comparative analysis of five plastid genomes of Apiaceae (Downie & Jansen, 2015). In studies of other families, these aforementioned loci have previously been considered useful in resolving relationships. The *acc*D/*psa*I region was used in studies of *Pyrus* (Rosaceae), *Dendrochilum* (Orchidaceae), and *Castanea* (Fagaceae; Shaw, Lickey, Shilling, & Small, 2007). The *psb*Z/*trn*G region was found most divergent in the analysis of 13 *Gossypium* plastid genomes (Malvaceae; Xu et al., 2012). The *rps*16/*trn*Q region included many phylogenetically informative indels in *Carphephorus *(Asteraceae), *Gratiola *(Plantaginaceae), *Prunus* (Rosaceae), and *Trillium* (Melanthiaceae; Shaw et al., 2007), and the *trn*L intron resolved relationships in Gentianaceae (Yuan et al., 2003).

### 4.2 Novel DNA

The three *Prangos* species examined herein have each incorporated novel DNA into the LSC region adjacent to the LSC/IRa junction, with BLAST searches revealing 100–102 bp matches having ≥86% sequence identity to an IGS region adjacent to *Daucus* mitochondrial gene *cob*. The introgression of mtDNA into the plastid genome is not without precedence in Apiaceae, for previous studies have also reported the presence of mtDNA in the plastomes of some Apiaceae species (Downie & Jansen, 2015; Goremykin, Salamini, Velasco, & Viola, 2009; Iorizzo, Grzebelus, et al., 2012a; Iorizzo, Senalik, et al., 2012b; Peery, Downie, Jansen, & Raubeson, 2011). In *P. crispum*, for example, 345 bp of novel noncoding DNA has been incorporated into the LSC region adjacent to the LSC/IRb boundary and BLAST searches querying this insert resulted in hits of a 122 bp region to angiosperm mtDNA (Downie & Jansen, 2015). A comparison of IRa/LSC boundaries from 34 species of Apiaceae revealed that those species with their LSC/IRb boundaries within *rpl*2 have insertions suggestive of mtDNA, ranging in size from 40 to 447 bp, between IRa and *trn*H‐GUC (Peery, 2015).

Nevertheless, we interpret these results cautiously, for in the absence of complete mitochondrial genomes from *Prangos* and other related Apiaceae, the mitochondrial provenance of this novel DNA in *Prangos* is not absolute.

### 4.3 Utility of plastome SSRs and tandem repeat units

In plastomes of *D. carota* and other Apiaceae species (Peery, 2015; Ruhlman et al., 2006), the number of SSRs reported previously ranged from 44 to 55. In *Prangos* plastomes, the number of mono‐ and dinucleotide repeats is similar, varying between 48 (*P. lipskyi*) and 49 (*P. fedtschenkoi*). SSRs are widely used molecular markers in plant population and phylogenetic studies due to their high mutation rates and high levels of polymorphism among individuals of a population. Another advantage is their ease of use and low‐cost detection using PCR (Hoshino, Bravo, Nobile, & Morelli, 2012; Wang, Barkley, & Jenkins, 2009). To date, the availability of complete plastome sequences in public databases has reduced the economic costs of obtaining these data and increased their use in inter‐ and intraspecific phylogenetic research, population study, and conservation activities (Wang et al., 2009). In addition, taxon‐specific primers, such as those generated herein for the *Prangos acc*D/*psa*I highly variable IGS region, will be useful to assess genetic differentiation among and within its populations.

Short sequence repeats that arise by chance in DNA sequences along with mutational changes can be presumably expanded by slipped‐strand mispairing events into longer tandem repeats (Levinson & Gutman, 1987). Transposon‐mediated insertions along with replication slippage were suggested as being responsible for generating direct and inverted long repeats (Hoshino et al., 2012; Palmer, 1991). Ten long tandem repeats were identified in *Prangos*, four of which are located within the same genomic regions and identical in both plastomes. The three others, namely two in *ycf*2 and one in *trn*S, were reported previously by Ruhlman et al. (2006) for *D. carota*. These repeats plus one palindromic tandem repeat in the *pet*N/*psb*M IGS region occur in the same locations and are shared by most of the Apiaceae and Araliaceae species surveyed.

### 4.4 Inversions

The shared possession of the same inversion is usually viewed as reliable evidence of common ancestry, although the presence of base substitutions within the region may cause misinterpretation (Kim & Lee, 2005). Large plastid inversions, as examples, have been suggested to be highly reliable phylogenetic markers (Graham, Reeves, Burns, & Olmstead, 2000; Jansen & Palmer, 1987; Raubeson & Jansen, 1992a, 1992b). In contrast, the utility of small inversions in phylogenetic inference is poor, because they are reported as often being homoplastic (Graham et al., 2000). In this study, we identified two small inversions in *Prango*s, both occurring in loop regions of *ndh*B and *pet*B introns that are homoplastic within Apiaceae.

Comparisons of sequences comprising these 5‐ and 9‐bp inversions in *ndh*B and *pet*B introns with 23 other species (16 genera) of Apiaceae (representing a diversity of tribes and other major clades within subfamily Apioideae; Downie et al., 2010) and 2 genera of Araliaceae revealed both identical direct and inverted motifs of these two small regions. In many additional taxa, 1–2 bp substitutions within these regions were also apparent. Both flip‐flop oriented forms of these sequences were revealed in the three *Prangos* species examined herein, whereas congeners of other genera shared identical forms of each sequence. The flip‐flop orientation of loop sequences in the plastid *trn*L/*trn*F region was also described for nine *Jasminum *species (Oleaceae; Kim & Lee, 2005). Two different accessions of *J. elegans *and single accessions of each of the other eight species were used in phylogenetic analyses. The accessions of *J. elegans *had different orientations of a loop sequence, with one orientation occurring in five other *Jasminum* species and the other orientation found in the remaining species*.* These sequence data may confound phylogenetic relationships unless one is cognizant that an inversion has taken place. Our future studies will survey for the presence of these inverted regions in other *Prangos* species.

In summary, the complete *Prangos* plastome sequences reported herein enhance the genomic information available for *Prangos* and will contribute to further studies of germplasm diversity, phylogeny, and phylogeography of these ecologically and economically important species. These data provide a valuable source of markers for future research at low taxonomic level and population genetics with further implementation in ex situ conservation strategies and restoration/regeneration programs.

## CONFLICT OF INTEREST

The authors declare that the research was conducted in the absence of any commercial or financial relationships that could be construed as a potential conflict of interest.

## AUTHOR CONTRIBUTION

FUM and KCh conceived and designated the study; DKYi and FUM conducted the molecular and computational analyses; SRD and FUM also performed analyses, interpreted the results, and wrote the manuscript; and ChHSh and KShT provided the necessary facilities, equipment, and chemicals, and helped with the field research.

## DATA ACCESSIBILITY

Complete plastid genomes: Genbank accessions KY652265 (*P. fedtschenkoi*) and KY652266 (*P. lipskyi*).
