# Supplementary figures and images for: A comparative analysis of complete plastid genomes from Prangos fedtschenkoi and Prangos lipskyi (Apiaceae)
Source: Ecol Evol. 2018 Dec 26;9(1):364–77. doi: 10.1002/ece3.4753 (PMC6342102; doi:10.1002/ece3.4753)

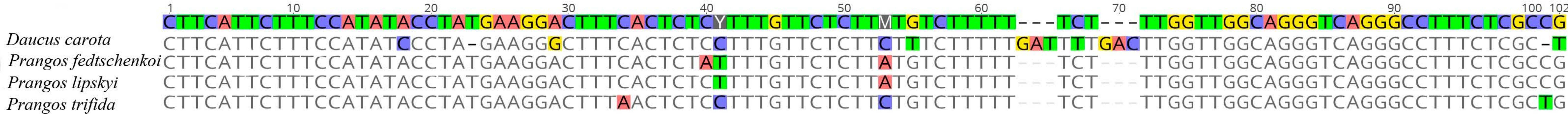

Supplement: Supplementary file 1 [file ECE3-9-364-s001.pdf]
