# Supplementary material for: A comparative analysis of complete plastid genomes from Prangos fedtschenkoi and Prangos lipskyi (Apiaceae)
Source: Ecol Evol. 2018 Dec 26;9(1):364–77. doi: 10.1002/ece3.4753 (PMC6342102; doi:10.1002/ece3.4753)
Supplement: Supplementary file 2 [file ECE3-9-364-s002.docx]

**Table S1.** The codon recognition pattern and codon usage for the *Prangos fedtschenkoi* chloroplast genome. Codons are presented by nucleotide triplets and their standard amino acid abbreviations; stop codons are identified by asterisks. Numerals indicate the frequency of usage of each codon.

| ATT | I | 971 | GTT | V | 506 | CGT | R | 317 | GTG | V | 188 |
| --- | --- | --- | --- | --- | --- | --- | --- | --- | --- | --- | --- |
| AAA | K | 926 | TCT | S | 504 | AAG | K | 304 | CTC | L | 177 |
| TTT | F | 896 | TTG | L | 499 | GAG | E | 293 | CTG | L | 158 |
| GAA | E | 889 | GTA | V | 485 | TCC | S | 288 | GTC | V | 157 |
| AAT | N | 847 | TTC | F | 451 | GGG | G | 287 | CCG | P | 156 |
| TTA | L | 827 | CAT | H | 436 | AAC | N | 277 | GCG | A | 155 |
| GAT | D | 751 | AGA | R | 418 | CCA | P | 276 | ACG | T | 141 |
| TAT | Y | 721 | ATC | I | 402 | ACC | T | 233 | AGG | R | 139 |
| ATA | I | 684 | TGG | W | 397 | GCC | A | 220 | CAC | H | 133 |
| GGA | G | 644 | CCT | P | 384 | TGT | C | 203 | CGG | R | 119 |
| CAA | Q | 618 | ACA | T | 377 | CAG | Q | 197 | AGC | S | 98 |
| GCT | A | 614 | GCA | A | 369 | TCG | S | 191 | CGC | R | 85 |
| GGT | G | 567 | AGT | S | 368 | CCC | P | 189 | TGC | C | 64 |
| ATG | M | 550 | CTA | L | 349 | TAC | Y | 189 | TAA | * | 42 |
| CTT | L | 542 | TCA | S | 335 | GAC | D | 188 | TAG | * | 25 |
| ACT | T | 528 | CGA | R | 322 | GGC | G | 188 | TGA | * | 17 |

**Table S2.** The codon recognition pattern and codon usage for the *Prangos lipskyi* chloroplast genome. Codons are presented by nucleotide triplets and their standard amino acid abbreviations; stop codons are identified by asterisks. Numerals indicate the frequency of usage of each codon.

| ATT | I | 1003 | TCT | S | 518 | CGT | R | 329 | GGC | G | 190 |
| --- | --- | --- | --- | --- | --- | --- | --- | --- | --- | --- | --- |
| AAA | K | 981 | GTT | V | 517 | AAG | K | 321 | CTC | L | 188 |
| GAA | E | 944 | TTG | L | 514 | GAG | E | 300 | CTG | L | 165 |
| TTT | F | 942 | GTA | V | 499 | GGG | G | 299 | GTC | V | 164 |
| AAT | N | 884 | TTC | F | 466 | TCC | S | 296 | CCG | P | 159 |
| TTA | L | 846 | CAT | H | 449 | AAC | N | 288 | GCG | A | 157 |
| GAT | D | 779 | AGA | R | 440 | CCA | P | 277 | ACG | T | 148 |
| TAT | Y | 746 | ATC | I | 416 | ACC | T | 243 | AGG | R | 144 |
| ATA | I | 706 | TGG | W | 407 | GCC | A | 224 | CAC | H | 140 |
| GGA | G | 664 | CCT | P | 404 | TGT | C | 208 | CGG | R | 122 |
| CAA | Q | 627 | ACA | T | 393 | TCG | S | 203 | AGC | S | 102 |
| GCT | A | 618 | AGT | S | 383 | CAG | Q | 202 | CGC | R | 88 |
| GGT | G | 579 | GCA | A | 376 | TAC | Y | 199 | TGC | C | 68 |
| ATG | M | 568 | CTA | L | 370 | GAC | D | 195 | TAA | * | 42 |
| CTT | L | 552 | TCA | S | 351 | CCC | P | 195 | TAG | * | 25 |
| ACT | T | 540 | CGA | R | 339 | GTG | V | 192 | TGA | * | 16 |

**Table S3.** Comparison of base substitutions and indels in protein coding genes between *Prangos fedtschenkoi* (*P.f.*) and *Prangos lipskyi* (*P.l.*) plastomes

| Region | Gene | Size (bp) | | Aligned length (bp) | Indel  (bp) | Number of indel events | Number of polymorphic sites | | | Nucleotide diversity | *Ks* | *Ka* | *Ka*/*Ks* | Identity (%) | Diversity  (%) |
| --- | --- | --- | --- | --- | --- | --- | --- | --- | --- | --- | --- | --- | --- | --- | --- |
|  |  | (*P.f.*) | (*P.l.*) |  |  |  | Total | Ts | Tv |  |  |  |  |  |  |
| LSC & IR | *rps*12 | 372 | 372 | 372 |  |  | 1 |  | 1 | 0.00269 | 0 | 0.0037 |  | 99.7 | 0.3 |
| LSC | *psb*A | 1062 | 1062 | 1062 |  |  | 1 |  | 1 | 0.00282 | 0 | 0.0036 |  | 99.7 | 0.3 |
| LSC | *mat*K | 1557 | 1557 | 1557 |  |  | 5 |  | 5 | 0.00639 | 0.0091 | 0.0057 | 0.6264 | 99.4 | 0.6 |
| LSC | *rps*16 | 237 | 237 | 237 |  |  | 1 |  | 1 | 0.00422 | 0 | 0.0055 |  | 99.6 | 0.4 |
| LSC | *psb*K | 186 | 186 | 186 |  |  | 0 |  |  | 0 | 0 | 0 |  | 100 | 0 |
| LSC | *psb*I | 111 | 111 | 111 |  |  | 1 | 1 |  | 0.00909 | 0.0091 | 0 | 0 | 99.1 | 0.9 |
| LSC | *atp*A | 1524 | 1524 | 1524 |  |  | 4 | 4 |  | 0.0026 | 0.0026 | 0 | 0 | 99.7 | 0.3 |
| LSC | *atp*F | 546 | 546 | 546 |  |  | 1 |  | 1 | 0.00249 | 0.0025 | 0 | 0 | 99.8 | 0.2 |
| LSC | *atp*H | 246 | 246 | 246 |  |  | 0 |  |  | 0 | 0 | 0 |  | 100 | 0 |
| LSC | *atp*I | 744 | 744 | 744 |  |  | 4 | 3 | 1 | 0.00538 | 0.0054 | 0 | 0 | 99.5 | 0.5 |
| LSC | *rps*2 | 711 | 711 | 711 |  |  | 0 |  |  | 0 | 0 | 0 |  | 100 | 0 |
| LSC | *rpo*C2 | 4161 | 4161 | 4161 |  |  | 2 | 1 | 1 | 0.00168 | 0.0034 | 0.0012 | 0.3529 | 99.8 | 0.2 |
| LSC | *rpo*C1 | 2058 | 2058 | 2058 |  |  | 2 | 1 | 1 | 0.00284 | 0 | 0.0008 |  | 99.9 | 0.1 |
| LSC | *rpo*B | 3213 | 3213 | 3213 |  |  | 6 | 3 | 3 | 0.00218 | 0.0015 | 0.0024 | 1.6 | 99.8 | 0.2 |
| LSC | *pet*N | 90 | 90 | 90 |  |  | 0 |  |  | 0 | 0 | 0 |  | 100 | 0 |
| LSC | *psb*M | 117 | 117 | 117 |  |  | 0 |  |  | 0 | 0 | 0 |  | 100 | 0 |
| LSC | *psb*D | 1062 | 1062 | 1062 |  |  | 0 |  |  | 0 | 0 | 0 |  | 100 | 0 |
| LSC | *psb*C | 1422 | 1422 | 1422 |  |  | 1 | 1 |  | 0.0007 | 0 | 0.0009 |  | 99.9 | 0.1 |
| LSC | *psb*Z | 189 | 189 | 189 |  |  | 0 |  |  | 0 | 0 | 0 |  | 100 | 0 |
| LSC | *rps*14 | 303 | 303 | 303 |  |  | 0 |  |  | 0 | 0 | 0 |  | 100 | 0 |
| LSC | *psa*B | 2205 | 2205 | 2205 |  |  | 4 | 2 | 2 | 0.00182 | 0 | 0.0023 |  | 99.8 | 0.2 |
| LSC | *psa*A | 2253 | 2253 | 2253 |  |  | 2 | 2 |  | 0.00089 | 0 | 0.0011 |  | 99.9 | 0.1 |
| LSC | *ycf*3 | 507 | 507 | 507 |  |  | 0 |  |  | 0 | 0 | 0 |  | 100 | 0 |
| LSC | *rps*4 | 606 | 606 | 606 |  |  | 4 | 4 |  | 0 | 0 | 0.0084 |  | 99.3 | 0.7 |
| LSC | *ndh*J | 477 | 477 | 477 |  |  | 0 |  |  | 0 | 0 | 0 |  | 100 | 0 |
| LSC | *ndh*K | 678 | 678 | 678 |  |  | 0 |  |  | 0 | 0 | 0 |  | 100 | 0 |
| LSC | *ndh*C | 363 | 363 | 363 |  |  | 0 |  |  | 0 | 0 | 0 |  | 100 | 0 |
| LSC | *atp*E | 423 | 423 | 423 |  |  | 0 |  |  | 0 | 0 | 0 |  | 100 | 0 |
| LSC | *atp*B | 1497 | 1497 | 1497 |  |  | 1 | 1 |  | 0.00067 | 0 | 0.0008 |  | 99.9 | 0.1 |
| LSC | *rbc*L | 1428 | 1428 | 1428 |  |  | 3 | 3 |  | 0.00210 | 0.0087 | 0 | 0 | 99.8 | 0.2 |
| LSC | *acc*D | 1470 | 1470 | 1470 |  |  | 2 |  | 2 | 0.00136 | 0 | 0.0017 |  | 99.9 | 0.1 |
| LSC | *psa*I | 111 | 111 | 111 |  |  | 0 |  |  | 0 | 0 | 0 |  | 100 | 0 |
| LSC | *ycf*4 | 555 | 555 | 555 |  |  | 0 |  |  | 0 | 0 | 0 |  | 100 | 0 |
| LSC | *cem*A | 690 | 690 | 690 |  |  | 1 |  | 1 | 0.00145 | 0 | 0.0019 |  | 99.9 | 0.1 |
| LSC | *pet*A | 963 | 963 | 963 |  |  | 1 |  | 1 | 0.00104 | 0.0043 | 0 | 0 | 99.9 | 0.1 |
| LSC | *psb*J | 123 | 123 | 123 |  |  | 0 |  |  | 0 | 0 | 0 |  | 100 | 0 |
| LSC | *psb*L | 117 | 117 | 117 |  |  | 0 |  |  | 0 | 0 | 0 |  | 100 | 0 |
| LSC | *psb*F | 120 | 120 | 120 |  |  | 0 |  |  | 0 | 0 | 0 |  | 100 | 0 |
| LSC | *psb*E | 252 | 252 | 252 |  |  | 1 | 1 |  | 0.03970 | 0 | 0.0051 |  | 99.6 | 0.4 |
| LSC | *pet*L | 96 | 96 | 96 |  |  | 0 |  |  | 0 | 0 | 0 |  | 100 | 0 |
| LSC | *pet*G | 114 | 114 | 114 |  |  | 0 |  |  | 0 | 0 | 0 |  | 100 | 0 |
| LSC | *psa*J | 129 | 129 | 129 |  |  | 0 |  |  | 0 | 0 | 0 |  | 100 | 0 |
| LSC | *rpl*33 | 201 | 201 | 201 |  |  | 0 |  |  | 0 | 0 | 0 |  | 100 | 0 |
| LSC | *rps*18 | 306 | 306 | 306 |  |  | 2 | 2 |  | 0.00650 | 0.0134 | 0.0044 | 0.3283 | 99.3 | 0.7 |
| LSC | *rpl*20 | 387 | 387 | 387 |  |  | 0 |  |  | 0 | 0 | 0 |  | 100 | 0 |
| LSC | *clp*P | 594 | 594 | 594 |  |  | 2 | 1 | 1 | 0.00866 | 0 | 0.0109 |  | 99.7 | 0.3 |
| LSC | *psb*B | 1527 | 1527 | 1527 |  |  | 3 | 2 | 1 | 0.00196 | 0.0054 | 0.0009 | 0.1666 | 99.8 | 0.2 |
| LSC | *psb*T | 102 | 102 | 102 |  |  | 1 |  | 1 | 0.00980 | 0.038 | 0 | 0 | 99 | 1 |
| LSC | *psb*N | 132 | 132 | 132 |  |  | 0 |  |  | 0 | 0 | 0 |  | 100 | 0 |
| LSC | *psb*H | 222 | 222 | 222 |  |  | 0 |  |  | 0 | 0 | 0 |  | 100 | 0 |
| LSC | *pet*B | 648 | 648 | 648 |  |  | 0 |  |  | 0 | 0 | 0 |  | 100 | 0 |
| LSC | *pet*D | 486 | 486 | 486 |  |  | - |  |  | 0 | 0 | 0 |  | 100 | 0 |
| LSC | *rpo*A | 1005 | 1062 | 1010 | 6; 51 | 2 | 7 | 5 | 2 | 0.00697 | 0.0183 | 0.0026 | 0.1420 | 98.7 | 1.3 |
| LSC | *rps*11 | 417 | 417 | 417 |  |  | 0 |  |  | 0 | 0 | 0 |  | 100 | 0 |
| LSC | *rpl*36 | 114 | 114 | 114 |  |  | 0 |  |  | 0 | 0 | 0 |  | 100 | 0 |
| LSC | *inf*A | 234 | 234 | 234 |  |  | 0 |  |  | 0 | 0 | 0 |  | 100 | 0 |
| LSC | *rps*8 | 405 | 405 | 405 |  |  | 1 | 1 |  | 0 | 0 | 0.0032 |  | 99.8 | 0.2 |
| LSC | *rpl*14 | 369 | 369 | 369 |  |  | 1 |  | 1 | 0.00270 | 0 | 0.0035 |  | 99.7 | 0.3 |
| LSC | *rpl*16 | 408 | 408 | 408 |  |  | 0 |  |  | 0 | 0 | 0 |  | 100 | 0 |
| LSC | *rps*3 | 657 | 657 | 657 |  |  | 2 | 1 | 1 | 0.00304 | 0.0076 | 0.0019 | 0.25 | 99.7 | 0.3 |
| LSC | *rpl*22 | 495 | 495 | 495 |  |  | 1 |  | 1 | 0.00202 | 0 | 0.0025 |  | 99.8 | 0.2 |
| LSC | *rps*19 | 279 | 279 | 279 |  |  | 0 |  |  | 0 | 0 | 0 |  | 100 | 0 |
| **LSC TOTAL** | | **44076** | **44133** | **44081** | **57** | **2** | **68** | **39** | **29** | **0.0022** | **0.0021** | **0.0012** | **0.2476** | **99.8** | **0.2** |
| IR | *rpl*2 | 825 | 825 | 825 |  |  | 1 | 1 |  | 0.00230 | 0.0095 | 0 | 0 | 99.9 | 0.1 |
| IR | *rpl*23 | 282 | 282 | 282 |  |  | 0 |  |  | 0 | 0 | 0 |  | 100 | 0 |
| IR | *ycf*2 | 6297 | 6297 | 6297 |  |  | 4 | 3 | 1 | 0.00064 | 0.0007 | 0.0006 | 0.8572 | 99.9 | 0.1 |
| IR | *ndh*B | 1533 | 1533 | 1533 |  |  | 0 |  |  | 0 | 0 | 0 |  | 100 | 0 |
| IR | *rps*7 | 468 | 468 | 468 |  |  | 0 |  |  | 0 | 0 | 0 |  | 100 | 0 |
| IR | *rrn*16 | 1491 | 1491 | 1491 |  |  | 1 | 1 |  | 0.00067 | 0.0027 | 0 | 0 | 99.9 | 0.1 |
| IR | *rrn*23 | 2814 | 2814 | 2814 |  |  | 1 | 1 |  | 0.0061 | 0 | 0 |  | 99.9 | 0.1 |
| IR | *rrn*4.5 | 103 | 103 | 103 |  |  | 0 |  |  | 0 | 0 | 0 |  | 100 | 0 |
| IR | *rrn*5 | 121 | 121 | 121 |  |  | 0 |  |  | 0 | 0 | 0 |  | 100 | 0 |
| **IR TOTAL** | | **13934** | **13934** | **13934** | **0** | **0** | **7** | **6** | **1** | **0.0011** | **0.0014** | **0.0001** | **0.2857** | **99.9** | **0.1** |
| SSC | *ndh*F | 2247 | 2253 | 2253 | 6 | 1 | 3 |  | 3 | 0.00134 | 0 | 0.0017 |  | 99.8 | 0.2 |
| SSC | *rpl*32 | 162 | 162 | 162 |  |  | 1 | 1 |  | 0.00617 | 0.0258 | 0 | 0 | 99.4 | 0.6 |
| SSC | *ccs*A | 975 | 975 | 975 |  |  | 2 | 2 |  | 0.00205 | 0.009 | 0 | 0 | 99.8 | 0.2 |
| SSC | *ndh*D | 1530 | 1530 | 1530 |  |  | 5 | 3 | 2 | 0.00333 | 0 | 0.0041 |  | 99.8 | 0.2 |
| SSC | *psa*C | 246 | 246 | 246 |  |  | 0 |  |  | 0 | 0 | 0 |  | 100 | 0 |
| SSC | *ndh*E | 306 | 306 | 306 |  |  | 1 |  | 1 | 0.00327 | 0 | 0.0043 |  | 99.7 | 0.3 |
| SSC | *ndh*G | 531 | 531 | 531 |  |  | 0 |  |  | 0 | 0 | 0 |  | 100 | 0 |
| SSC | *ndh*I | 504 | 504 | 504 |  |  | 0 |  |  | 0 | 0 | 0 |  | 100 | 0 |
| SSC | *ndh*A | 1092 | 1092 | 1092 |  |  | 2 | 1 | 1 | 0.00372 | 0 | 0.0046 |  | 99.8 | 0.2 |
| SSC | *ndh*H | 1182 | 1182 | 1182 |  |  | 5 | 1 | 4 | 0.00423 | 0.0042 | 0.0043 |  | 99.6 | 0.4 |
| SSC | *rps*15 | 273 | 273 | 273 |  |  | 1 |  | 1 | 0.00366 | 0 | 0.0046 |  | 99.6 | 0.4 |
| SSC | *ycf*1 | 5484 | 5493 | 5496 | 3; 12 | 2 | 26 | 9 | 17 | 0.00477 | 0.0026 | 0.0053 | 2.03846 | 99.3 | 0.7 |
| **SSC TOTAL** | | **14532** | **14547** | **14550** | **21** | **3** | **46** | **17** | **29** | **0.0027** | **0.0035** | **0.0024** | **0.6795** | **99.7** | **0.3** |
| **TOTAL** | | **72542** | **72614** | **72565** | **78** | **5** | **121** | **62** | **59** | **0.0020** | **0.0024** | **0.0012** | **0.3988** | **99.8** | **0.2** |

Ts = transitions; Tv = transversions; *Ks* = the number of synonymous substitutions per synonymous site; *K*a = the number of nonsynonymous substitutions per nonsynonymous site; LSC = large single copy; IR = inverted repeat; SSC = small single copy.

**Table S4**. Comparison of base substitutions and indels in intergenic sequences between *Prangos fedtschenkoi* (*P.f.*) and *Prangos lipskyi* (*P.l.*) plastomes

| Region | IGS | Size (bp) | | Aligned  length  (bp) | Indel  (bp) | Number of indel events | Number of polymorphic sites | | | Nucleotide  diversity | Identity  (%) | Diversity  (%) |
| --- | --- | --- | --- | --- | --- | --- | --- | --- | --- | --- | --- | --- |
|  |  | (*P.f.*) | (*P.l*.) |  |  |  | Total | Ts | Tv |  |  |  |
| LSC | *trn*H-GUG/*psb*A | 192 | 192 | 193 | 1; 1 | 2 | 2 |  | 2 | 0.01047 | 97.9 | 2.1 |
| LSC | *psb*A/*trn*K-UUU | 214 | 211 | 214 | 3 | 1 | 2 | 1 | 1 | 0.01420 | 97.2 | 2.8 |
| LSC | *trn*K-UUU/*mat*K | 229 | 229 | 229 |  |  | 1 |  | 1 | 0.00437 | 99.6 | 0.4 |
| LSC | *mat*K/*trn*K-UUU | 723 | 723 | 723 |  |  | 4 | 1 | 3 | 0.00553 | 99.4 | 0.6 |
| LSC | *trn*K-UUU/*rps*16 | 729 | 751 | 756 | 1; 1; 1; 1; 2; 6; 9; 11 | 8 | 1 |  | 1 | 0.00139 | 95.6 | 4.4 |
| LSC | *rps*16/*trn*Q-UUG | 1185 | 1226 | 1257 | 3; 14; 17; 69 | 4 | 4 | 1 | 3 | 0.00347 | 91.5 | 8.5 |
| LSC | *trn*Q-UUG/*psb*K | 351 | 351 | 351 |  |  | 2 | 2 |  | 0.00570 | 99.4 | 0.6 |
| LSC | *psb*K/*psb*I | 324 | 323 | 324 | 1 | 1 | 2 | 2 |  | 0.00621 | 99.1 | 0.9 |
| LSC | *psb*I/*trn*S-GCU | 140 | 140 | 140 |  |  | 0 |  |  | 0 | 100 | 0 |
| LSC | *trn*S-GCU/*trn*G-GCC | 554 | 553 | 555 | 1; 2 | 2 | 2 | 1 | 1 | 0.00362 | 99.1 | 0.9 |
| LSC | *trn*G-GCC/*trn*R-UCU | 152 | 159 | 159 | 7 | 1 | 0 |  |  | 0 | 95.6 | 4.4 |
| LSC | *trn*R-UCU/*atp*A | 108 | 110 | 110 | 2 | 1 | 0 |  |  | 0 | 98.2 | 1.8 |
| LSC | *atp*A/*atp*F | 52 | 54 | 54 | 2 | 1 | 0 |  |  | 0 | 96.3 | 3.7 |
| LSC | *atp*F/*atp*H | 281 | 281 | 281 |  |  | 0 |  |  | 0 | 100 | 0 |
| LSC | *atp*H/*atp*I | 773 | 765 | 773 | 1; 7 | 2 | 3 | 3 |  | 0.00390 | 98.6 | 1.4 |
| LSC | *atp*I/*rps*2 | 265 | 273 | 273 | 8 | 1 | 0 |  |  | 0 | 97.1 | 2.9 |
| LSC | *rps*2/*rpo*C2 | 236 | 233 | 236 | 3 | 1 | 3 | 3 |  | 0.00858 | 97.5 | 2.5 |
| LSC | *rpo*C2/*rpo*C1 | 211 | 211 | 211 |  |  | 0 |  |  | 0 | 100 | 0 |
| LSC | *rpo*C1/*rpo*B | 5 | 5 | 5 |  |  | 0 |  |  | 0 | 100 | 0 |
| LSC | *rpo*B/*trn*C-GCA | 1236 | 1228 | 1247 | 1; 5; 10; 14 | 4 | 4 | 1 | 3 | 0.00329 | 97.3 | 2.7 |
| LSC | *trn*C-GCA/*pet*N | 684 | 679 | 684 | 5 | 1 | 3 | 1 | 2 | 0.00442 | 98.8 | 1.2 |
| LSC | *pet*N/*psb*M | 1116 | 1132 | 1132 | 2; 14 | 2 | 8 | 3 | 5 | 0.00717 | 97.9 | 2.1 |
| LSC | *psb*M/*trn*D-GUC | 688 | 689 | 689 | 1 | 1 | 1 | 1 |  | 0.00145 | 99.7 | 0.3 |
| LSC | *trn*D-GUC/*trn*Y-GUA | 121 | 121 | 121 |  |  | 1 |  | 1 | 0.00826 | 99.2 | 0.8 |
| LSC | *trn*Y-GUA/*trn*E-UUC | 76 | 76 | 76 |  |  | 1 | 1 |  | 0.01316 | 98.7 | 1.3 |
| LSC | *trn*E-UUC/*trn*T-GGU | 691 | 695 | 699 | 1; 4; 7 | 3 | 7 | 4 | 3 | 0.01592 | 97.3 | 2.7 |
| LSC | *trn*T-GGU/*psb*D | 1434 | 1443 | 1444 | 1; 1; 2; 7 | 4 | 7 | 1 | 6 | 0.00488 | 98.8 | 1.2 |
| LSC | *psb*D/*psb*C | - | - | - |  |  | 0 |  |  | - | - |  |
| LSC | *psb*C/*trn*S-UGA | 242 | 247 | 247 | 1; 4 | 2 | 1 |  | 1 | 0.00413 | 97.6 | 2.4 |
| LSC | *trn*S-UGA/*psb*Z | 359 | 352 | 359 | 1; 6 | 3 | 3 | 1 | 2 | 0.00852 | 97.2 | 2.8 |
| LSC | *psb*Z/*trn*G-GCC | 296 | 330 | 334 | 1; 3; 38 | 3 | 2 | 2 |  | 0.00685 | 86.8 | 13.2 |
| LSC | *trn*G-GCC/*trn*fM-CAU | 180 | 180 | 180 |  |  | 1 | 1 |  | 0.00556 | 99.4 | 0.6 |
| LSC | *trn*fM-CAU/*rps*14 | 164 | 164 | 164 |  |  | 1 | 1 |  | 0.00610 | 99.4 | 0.6 |
| LSC | *rps*14/*psa*B | 123 | 114 | 114 | 9 | 1 | 0 |  |  | 0 | 92.7 | 7.3 |
| LSC | *psa*B/*psa*A | 25 | 25 | 25 |  |  | 0 |  |  | 0 | 100 | 0 |
| LSC | *psa*A/*ycf*3 | 703 | 702 | 703 | 1 | 1 | 3 | 2 | 1 | 0.00427 | 99.4 | 0.6 |
| LSC | *ycf*3/*trn*S-GGA | 818 | 822 | 824 | 1; 1; 1; 5 | 4 | 4 | 2 | 2 | 0.00490 | 98.5 | 1.5 |
| LSC | *trn*S-GGA/*rps*4 | 299 | 299 | 299 |  |  | 0 |  |  | 0 | 100 | 0 |
| LSC | *rps*4/*trn*T-UGU | 366 | 385 | 385 | 19 | 1 | 3 |  | 3 | 0.00820 | 94.3 | 5.7 |
| LSC | *trn*T-UGU/*trn*L-UAA | 804 | 830 | 830 | 1; 5; 9; 11 | 4 | 5 | 2 | 3 | 0.00622 | 96.3 | 3.7 |
| LSC | *trn*L-UAA/*trn*F-GAA | 363 | 370 | 370 | 7 | 1 | 0 |  |  | 0 | 98.1 | 1.9 |
| LSC | *trn*F-GAA/*ndh*J | 355 | 355 | 355 |  |  | 3 |  | 3 | 0 | 99.2 | 0.8 |
| LSC | *ndh*J/*ndh*K | 116 | 116 | 116 |  |  | 1 |  | 1 | 0.00862 | 99.1 | 0.9 |
| LSC | *ndh*K/*ndh*C | 48 | 46 | 48 | 2 | 1 | 0 |  |  | 0 | 95.8 | 4.2 |
| LSC | *ndh*C/*trn*V-UAC | 1115 | 1125 | 1128 | 3; 13 | 2 | 2 |  | 2 | 0 | 98.3 | 1.7 |
| LSC | *trn*V-UAC/*trn*M-CAU | 175 | 175 | 175 |  |  | 0 |  |  | 0 | 100 | 0 |
| LSC | *trn*M-CAU/*atp*E | 190 | 187 | 190 | 3 | 1 | 1 | 1 |  | 0.00535 | 97.9 | 2.1 |
| LSC | *atp*E/*atp*B | - | - | - |  |  | 0 |  |  |  |  |  |
| LSC | *atp*B/*rbc*L | 760 | 759 | 761 | 1; 2 | 2 | 3 | 1 | 2 | 0.00396 | 99.2 | 0.8 |
| LSC | *rbc*L/*acc*D | 602 | 602 | 602 |  |  | 3 |  | 3 | 0.00499 | 99.5 | 0.5 |
| LSC | *acc*D/*psa*I | 499 | 574 | 596 | 6; 11; 11; 34; 57 | 5 | 7 | 2 | 5 | 0.02213 | 78.9 | 21.1 |
| LSC | *psa*I/*ycf*4 | 397 | 402 | 402 | 5 | 1 | 0 |  |  | 0 | 98.8 | 1.2 |
| LSC | *ycf*4/*cem*A | 693 | 691 | 693 | 2 | 1 | 0 |  |  | 0 | 99.7 | 0.3 |
| LSC | *cem*A/*pet*A | 245 | 238 | 245 | 7 | 1 | 0 |  |  | 0 | 97.1 | 2.9 |
| LSC | *pet*A/*psb*J | 635 | 661 | 661 | 3; 23 | 2 | 2 |  | 2 | 0.00315 | 95.8 | 4.2 |
| LSC | *psb*J/*psb*L | 132 | 132 | 132 |  |  | 0 |  |  | 0 | 100 | 0 |
| LSC | *psb*L/*psb*F | 22 | 22 | 22 |  |  | 0 |  |  | 0 | 100 | 0 |
| LSC | *psb*F/*psb*E | 9 | 9 | 9 |  |  | 0 |  |  | 0 | 100 | 0 |
| LSC | *psb*E/*pet*L | 1010 | 1010 | 1010 |  |  | 4 | 2 | 2 | 0.00396 | 99.6 | 0.4 |
| LSC | *pet*L/*pet*G | 152 | 152 | 152 |  |  | 0 |  |  | 0 | 100 | 0 |
| LSC | *pet*G/*trn*W-CCA | 129 | 129 | 129 |  |  | 0 |  |  | 0 | 100 | 0 |
| LSC | *trn*W-CCA/*trn*P-UGG | 136 | 136 | 136 |  |  | 1 | 1 |  | 0.00735 | 99.3 | 0.7 |
| LSC | *trn*P-UGG/*psa*J | 386 | 386 | 386 |  |  | 0 |  |  | 0 | 100 | 0 |
| LSC | *psa*J/*rpl*33 | 462 | 458 | 462 | 4 | 1 | 4 | 1 | 3 | 0.00873 | 98.3 | 1.7 |
| LSC | *rpl*33/*rps*18 | 185 | 185 | 185 |  |  | 2 |  | 2 | 0.01081 | 98.9 | 1.1 |
| LSC | *rps*18/*rpl*20 | 233 | 234 | 234 | 1 | 1 | 0 |  |  | 0 | 99.6 | 0.4 |
| LSC | *rpl*20/*rps*12-2 | 770 | 769 | 770 | 1 | 1 | 1 |  | 1 | 0.00130 | 99.7 | 0.3 |
| LSC | *rps*12-2/*clp*P | 145 | 145 | 145 |  |  | 0 |  |  | 0 | 100 | 0 |
| LSC | *clp*P/*psb*B | 451 | 449 | 451 | 1; 1 | 2 | 0 |  |  | 0 | 99.6 | 0.4 |
| LSC | *psb*B/*psb*T | 210 | 200 | 210 | 10 | 1 | 0 |  |  | 0 | 95.2 | 4.8 |
| LSC | *psb*T/*psb*N | 86 | 86 | 86 |  |  | 0 |  |  | 0 | 100 | 0 |
| LSC | *psb*N/*psb*H | 102 | 102 | 102 |  |  | 1 | 1 |  | 0.00980 | 99 | 1 |
| LSC | *psb*H/*pet*B | 131 | 130 | 131 | 1 | 1 | 0 |  |  | 0 | 99.2 | 0.8 |
| LSC | *pet*B/*pet*D | 172 | 179 | 179 | 7 | 1 | 0 |  |  | 0 | 100 | 0 |
| LSC | *pet*D/*rpo*A | 184 | 133 | 133 | 51 | 1 | 1 |  | 1 | 0.01093 | 98.9 | 1.1 |
| LSC | *rpo*A/*rps*11 | 67 | 67 | 67 |  |  | 0 |  |  | 0 | 100 | 0 |
| LSC | *rps*11/*rpl*36 | 113 | 113 | 113 |  |  | 0 |  |  | 0 | 100 | 0 |
| LSC | *rpl*36/*inf*A | 116 | 117 | 117 | 1 | 1 | 2 |  | 2 | 0.01724 | 97.4 | 2.6 |
| LSC | *inf*A/*rps*8 | 121 | 121 | 121 |  |  | 0 |  |  | 0 | 100 | 0 |
| LSC | *rps*8/*rpl*14 | 201 | 202 | 202 | 1 | 1 | 3 | 3 |  | 0.01493 | 98 | 2 |
| LSC | *rpl*14/*rpl*16 | 132 | 132 | 132 |  |  | 1 | 1 |  | 0.00758 | 99.2 | 0.8 |
| LSC | *rpl*16/*rps*3 | 153 | 147 | 153 | 6 | 1 | 1 |  | 1 | 0.0068 | 95.4 | 4.6 |
|  | *rps*3/*rpl*22 | - | - | - |  |  | 0 |  |  |  |  |  |
| LSC | *rpl*22/*rps*19 | 66 | 66 | 66 |  |  | 0 |  |  | 0 | 100 | 0 |
| **LSC TOTAL** | | **29418** | **29615** | **29777** | **641** | **88** | **124** | **50** | **74** | **0.0041** | **98.1** | **1.9** |

**Table S4** (continued)

| Region | IGS | Size (bp) | | Aligned length  (bp) | Indel  (bp) | Number of indel events | Number of polymorphic sites | | | Nucleotide diversity | Identity  (%) | Diversity  (%) |
| --- | --- | --- | --- | --- | --- | --- | --- | --- | --- | --- | --- | --- |
|  |  | (*P.f.*) | (*P.l*.) |  |  |  | Total | Ts | Tv |  |  |  |
| IR | *rps*19/*rpl*2 | 58 | 54 | 58 | 4 | 1 | 0 |  |  | 0 | 93.1 | 6.9 |
| IR | *rpl*2/*rpl*23 | 18 | 18 | 18 |  |  | 0 |  |  | 0 | 100 | 0 |
| IR | *rpl*23/*trn*I-CAU | 165 | 165 | 165 |  |  | 1 |  | 1 | 0.00606 | 99.4 | 0.6 |
| IR | *trn*I-CAU/*ycf*2 | 88 | 88 | 88 |  |  | 0 |  |  | 0 | 100 | 0 |
| IR | *ycf*2/*trn*L-CAA | 586 | 586 | 586 |  |  | 0 |  |  | 0 | 100 | 0 |
| IR | *trn*L-CAA/*ndh*B | 573 | 573 | 573 |  |  | 0 |  |  | 0 | 100 | 0 |
| IR | *ndh*B/*rps*7 | 299 | 299 | 299 |  |  | 0 |  |  | 0 | 100 | 0 |
| IR | *rps*7/*rps*12-2 | 55 | 55 | 55 |  |  | 0 |  |  | 0 | 100 | 0 |
| IR | *rps*12-2/*trn*V-GAC | 1846 | 1847 | 1847 | 1 | 1 | 3 | 1 | 2 | 0.00163 | 99.8 | 0.2 |
| IR | trnV-GAC/*rrn*16 | 227 | 227 | 227 |  |  | 0 |  |  | 0 | 100 | 0 |
| IR | *rrn*16/*trn*I-GAU | 294 | 294 | 294 |  |  | 0 |  |  | 0 | 100 | 0 |
| IR | *trn*I-GAU/*trn*A-UGC | 64 | 64 | 64 |  |  | 0 |  |  | 0 | 100 | 0 |
| IR | *trn*A-UGC/*rrn*23 | 152 | 152 | 152 |  |  | 0 |  |  | 0 | 100 | 0 |
| IR | *rrn*23/*rrn*4.5 | 98 | 98 | 98 |  |  | 0 |  |  | 0 | 100 | 0 |
| IR | *rrn*4.5/*rrn*5 | 257 | 257 | 257 |  |  | 0 |  |  | 0 | 100 | 0 |
| IR | *rrn*5/*trn*R-ACG | 257 | 257 | 257 |  |  | 0 |  |  | 0 | 100 | 0 |
| IR | *trn*R-ACG/*trn*N-GUU | 583 | 583 | 584 | 1; 1 | 2 | 0 |  |  | 0 | 99.7 | 0.3 |
| IR | *trn*N-GUU/*ycf*1 | 327 | 327 | 327 |  |  | 1 |  | 1 | 0.00305 | 99.7 | 0.3 |
| **IR TOTAL** | | **5947** | **5944** | **5949** | **7** | **4** | **5** | **1** | **4** | **0.0006** | **99.54** | **0.46** |
| SSC | *ndh*F/*rpl*32 | 937 | 940 | 960 | 1; 1; 1; 7; 13; 20 | 6 | 3 |  | 3 | 0.00327 | 95.2 | 4.8 |
| SSC | *rpl*32/*trn*L-UAG | 912 | 921 | 921 | 1; 2; 6 | 3 | 2 |  | 2 | 0.00219 | 98.8 | 1.2 |
| SSC | *trn*L-UAG/*ccs*A | 96 | 96 | 96 |  |  | 1 |  | 1 | 0.01042 | 99 | 1 |
| SSC | *ccs*A/*ndh*D | 223 | 223 | 223 |  |  | 2 | 2 |  | 0.00890 | 99.1 | 0.9 |
| SSC | *ndh*D/*psa*C | 88 | 88 | 88 |  |  | 0 |  |  | 0 | 100 | 0 |
| SSC | *psa*C/*ndh*E | 266 | 266 | 266 |  |  | 0 |  |  | 0 | 100 | 0 |
| SSC | *ndh*E/*ndh*G | 271 | 218 | 221 | 3; 50 | 2 | 1 |  | 1 | 0.00459 | 80.1 | 19.9 |
| SSC | *ndh*G/*ndh*I | 361 | 361 | 361 |  |  | 2 | 1 | 1 | 0.00554 | 99.4 | 0.6 |
| SSC | *ndh*I/*ndh*A | 93 | 93 | 93 |  |  | 1 |  | 1 | 0.01099 | 98.9 | 1.1 |
| SSC | *ndh*A/*ndh*H | 1 | 1 | 1 |  |  | 0 |  |  | 0 | 100 | 0 |
| SSC | *ndh*H/*rps*15 | 91 | 91 | 91 |  |  | 0 |  |  | 0 | 100 | 0 |
| SSC | *rps*15/*ycf*1 | 370 | 357 | 370 | 7; 7 | 2 | 4 | 2 | 2 | 0.01124 | 95.1 | 4.9 |
| **SSC TOTAL** | | **3709** | **3655** | **3691** | **119** | **13** | **16** | **5** | **11** | **0.0048** | **97.13** | **2.87** |
| **TOTAL** | | **39074** | **39214** | **39417** | **767** | **105** | **145** | **56** | **89** | **0.0032** | **98.3** | **1.7** |

Ts = transitions; Tv = transversions; LSC = large single copy; IR = inverted repeat; SSC = small single copy; “-“ = no integenic sequence between *psb*D and *psb*C genes.

**Table S5.** Comparison of base substitutions and indels in introns between *Prangos fedtschenkoi* (*P.f.*) and *Prangos lipskyi* (*P.l.*) plastomes

| Region | Intron | Size (bp) | | Aligned length  (bp) | Indel  (bp) | Number of indel events | Number of polymorphic sites | | | Nucleotide  diversity | Identity  (%) | Diversity  (%) |
| --- | --- | --- | --- | --- | --- | --- | --- | --- | --- | --- | --- | --- |
|  |  | (*P.f.*) | (*P.l*.) |  |  |  | Total | Ts | Tv |  |  |  |
| LSC | *rps*16 | 864 | 869 | 870 | 1; 1; 5 | 3 | 2 | 2 |  | 0.00232 | 99 | 1 |
| LSC | *trn*G-GCC | 710 | 709 | 710 | 1 | 1 | 3 | 1 | 2 | 0.00423 | 99.4 | 0.6 |
| LSC | *atp*F | 735 | 734 | 735 | 1 | 1 | 1 | 1 |  | 0.00136 | 99.7 | 0.3 |
| LSC | *rpo*C1 | 743 | 742 | 743 | 1 | 1 | 3 | 3 |  | 0.00403 | 99.5 | 0.5 |
| LSC | *ycf*3 | 788 | 793 | 798 | 1; 5; 9 | 3 | 2 |  | 2 | 0.00255 | 97.7 | 2.3 |
| LSC | *ycf*3 | 714 | 711 | 714 | 3 | 1 | 3 | 1 | 2 | 0.03880 | 99.2 | 0.8 |
| LSC | *trn*L-UAA | 520 | 493 | 525 | 5; 32 | 2 | 2 |  | 2 | 0.00410 | 92.6 | 7.4 |
| LSC | *trn*V-UAC | 567 | 566 | 567 | 1 | 1 | 1 |  | 1 | 0.00177 | 99.6 | 0.4 |
| LSC | *clp*P | 641 | 638 | 644 | 1; 1; 1; 1; 5 | 5 | 5 | 4 | 1 | 0.00945 | 97.7 | 2.3 |
| LSC | *clp*P | 843 | 838 | 845 | 1; 1; 1; 6 | 4 | 4 | 2 | 2 | 0.00478 | 98.5 | 1.5 |
| LSC | *pet*B | 750 | 750 | 750 |  |  | 10 | 2 | 8 | 0.01333 | 98.7 | 1.3 |
| LSC | *pet*D | 741 | 736 | 741 | 5 | 1 | 1 | 1 |  | 0.00135 | 99.2 | 0.8 |
| LSC | *rpl*16 | 948 | 927 | 948 | 4; 6; 11 | 3 | 5 | 2 | 3 | 0.00324 | 97.3 | 2.7 |
| **LSC total** | | **9564** | **9506** | **9590** | **110** | **26** | **42** | **19** | **23** | **0.00702** | **98.3** | **1.7** |
| IR | *rpl*2 | 650 | 652 | 652 | 2 | 1 | 1 |  | 1 | 0.00154 | 99.5 | 0.5 |
| IR | *ndh*B | 682 | 682 | 682 |  |  | 6 |  | 6 | 0.00880 | 99.1 | 0.9 |
| IR | *rps*12-2 | 536 | 536 | 536 |  |  | 0 |  |  | 0 | 100 | 0 |
| IR | *trn*I-GAU | 944 | 944 | 945 | 1; 1 | 2 | 1 | 1 |  | 0.00106 | 99.7 | 0.3 |
| IR | *trn*A-UGC | 812 | 814 | 814 | 2 | 1 | 1 |  | 1 | 0.00123 | 99.6 | 0.4 |
| **IR total** | | **3624** | **3628** | **3629** | **6** | **4** | **9** | **1** | **8** | **0.00252** | **99.6** | **0.4** |
| SSC | *ndh*A | 1080 | 1078 | 1080 | 2 | 1 | 2 | 2 |  | 0.00186 | 99.6 | 0.4 |
| **SSC total** | | **1080** | **1078** | **1080** | **2** | **1** | **2** | **2** | **0** | **0.00186** | **99.6** | **0.4** |
| **TOTAL** | | **14268** | **14212** | **14299** | **118** | **31** | **53** | **22** | **31** | **0.00380** | **99.2** | **0.8** |

Ts = transitions; Tv = transversions; LSC = large single copy; IR = inverted repeat; SSC = small single copy.

**Table S6.** Comparison of sequence length and nucleotide diversity in protein coding genes of *Prangos fedtschenkoi* (*P.f*.), *Prangos lipskyi* (*P.l*.) and *Prangos trifida* (*P.t*.) plastomes

| Region | Gene | Size (bp) | | | Aligned length (bp) | Nucleotide diversity | Pairwise identity (%) | Pairwise diversity  (%) |
| --- | --- | --- | --- | --- | --- | --- | --- | --- |
|  |  | (*P.f.*) | (*P.l.*) | (*P.t.)* |  |  |  |  |
| LSC & IR | *rps*12 | 372 | 372 | 372 | 372 | 0.01645 | 99.8 | 0.2 |
| LSC | *psb*A | 1062 | 1062 | 1062 | 1062 | 0.00251 | 99.7 | 0.3 |
| LSC | *mat*K | 1557 | 1557 | 1557 | 1557 | 0.00728 | 99.3 | 0.7 |
| LSC | *rps*16 | 237 | 237 | 237 | 237 | 0.00563 | 99.4 | 0.6 |
| LSC | *psb*K | 186 | 186 | 186 | 186 | 0.00717 | 99.3 | 0.7 |
| LSC | *psb*I | 111 | 111 | 111 | 111 | 0.00601 | 99.4 | 0.6 |
| LSC | *atp*A | 1524 | 1524 | 1524 | 1524 | 0.00219 | 99.8 | 0.2 |
| LSC | *atp*F | 546 | 546 | 546 | 545 | 0.00367 | 99.6 | 0.4 |
| LSC | *atp*H | 246 | 246 | 246 | 246 | 0 | 100 | 0 |
| LSC | *atp*I | 744 | 744 | 744 | 744 | 0.00358 | 99.6 | 0.4 |
| LSC | *rps*2 | 711 | 711 | 711 | 711 | 0.00375 | 99.6 | 0.4 |
| LSC | *rpo*C2 | 4161 | 4161 | 4161 | 4161 | 0.00336 | 99.7 | 0.3 |
| LSC | *rpo*C1 | 2058 | 2058 | 2058 | 2058 | 0.00194 | 99.8 | 0.2 |
| LSC | *rpo*B | 3213 | 3213 | 3213 | 3213 | 0.00373 | 99.6 | 0.4 |
| LSC | *pet*N | 90 | 90 | 90 | 90 | 0 | 100 | 0 |
| LSC | *psb*M | 117 | 117 | 117 | 117 | 0 | 100 | 0 |
| LSC | *psb*D | 1062 | 1062 | 1062 | 1062 | 0.00126 | 99.9 | 0.1 |
| LSC | *psb*C | 1422 | 1422 | 1422 | 1422 | 0.00281 | 99.7 | 0.3 |
| LSC | *psb*Z | 189 | 189 | 189 | 189 | 0 | 100 | 0 |
| LSC | *rps*14 | 303 | 303 | 303 | 303 | 0.0022 | 99.8 | 0.2 |
| LSC | *psa*B | 2205 | 2205 | 2205 | 2205 | 0.00212 | 99.8 | 0.2 |
| LSC | *psa*A | 2253 | 2253 | 2253 | 2253 | 0.00089 | 99.9 | 0.1 |
| LSC | *ycf*3 | 507 | 507 | 507 | 509 | 0 | 99.7 | 0.3 |
| LSC | *rps*4 | 606 | 606 | 606 | 606 | 0.0055 | 99.4 | 0.6 |
| LSC | *ndh*J | 477 | 477 | 477 | 477 | 0.0014 | 99.9 | 0.1 |
| LSC | *ndh*K | 678 | 678 | 678 | 678 | 0.00393 | 99.6 | 0.4 |
| LSC | *ndh*C | 363 | 363 | 363 | 363 | 0 | 100 | 0 |
| LSC | *atp*E | 423 | 423 | 423 | 423 | 0.00315 | 99.7 | 0.3 |
| LSC | *atp*B | 1497 | 1497 | 1497 | 1497 | 0.00223 | 99.8 | 0.2 |
| LSC | *rbc*L | 1428 | 1428 | 1428 | 1428 | 0.00327 | 99.7 | 0.3 |
| LSC | *acc*D | 1470 | 1470 | 1470 | 1470 | 0.00227 | 99.8 | 0.2 |
| LSC | *psa*I | 111 | 111 | 111 | 111 | 0 | 100 | 0 |
| LSC | *ycf*4 | 555 | 555 | 555 | 555 | 0 | 100 | 0 |
| LSC | *cem*A | 690 | 690 | 690 | 690 | 0.00483 | 99.5 | 0.5 |
| LSC | *pet*A | 963 | 963 | 963 | 963 | 0.00277 | 99.7 | 0.3 |
| LSC | *psb*J | 123 | 123 | 123 | 123 | 0 | 100 | 0 |
| LSC | *psb*L | 117 | 117 | 117 | 117 | 0.0057 | 99.4 | 0.6 |
| LSC | *psb*F | 120 | 120 | 120 | 120 | 0 | 100 | 0 |
| LSC | *psb*E | 252 | 252 | 252 | 252 | 0.00529 | 99.5 | 0.5 |
| LSC | *pet*L | 96 | 96 | 96 | 96 | 0 | 100 | 0 |
| LSC | *pet*G | 114 | 114 | 114 | 114 | 0 | 100 | 0 |
| LSC | *psa*J | 129 | 129 | 129 | 129 | 0 | 100 | 0 |
| LSC | *rpl*33 | 201 | 201 | 201 | 201 | 0.00332 | 99.7 | 0.3 |
| LSC | *rps*18 | 306 | 306 | 306 | 306 | 0.00871 | 99.1 | 0.9 |
| LSC | *rpl*20 | 387 | 387 | 387 | 387 | 0.00345 | 99.7 | 0.3 |
| LSC | *clp*P | 594 | 594 | 594 | 594 | 0.00337 | 99.7 | 0.3 |
| LSC | *psb*B | 1527 | 1527 | 1527 | 1527 | 0.00306 | 99.7 | 0.3 |
| LSC | *psb*T | 102 | 102 | 102 | 102 | 0.01307 | 98.7 | 1.3 |
| LSC | *psb*N | 132 | 132 | 132 | 132 | 0.00505 | 99.5 | 0.5 |
| LSC | *psb*H | 222 | 222 | 222 | 222 | 0 | 100 | 0 |
| LSC | *pet*B | 648 | 648 | 648 | 648 | 0.00412 | 99.6 | 0.4 |
| LSC | *pet*D | 486 | 486 | 489 | 489 | 0.00276 | 99.2 | 0.8 |
| LSC | *rpo*A | 1005 | 1062 | 1062 | 1062 | 0.00829 | 95.5 | 4.5 |
| LSC | *rps*11 | 417 | 417 | 417 | 417 | 0 | 100 | 0 |
| LSC | *rpl*36 | 114 | 114 | 114 | 114 | 0 | 100 | 0 |
| LSC | *inf*A | 234 | 234 | 234 | 234 | 0.00285 | 99.7 | 0.3 |
| LSC | *rps*8 | 405 | 405 | 405 | 405 | 0.00329 | 99.7 | 0.3 |
| LSC | *rpl*14 | 369 | 369 | 369 | 369 | 0.00542 | 99.5 | 0.5 |
| LSC | *rpl*16 | 408 | 408 | 408 | 408 | 0.00163 | 99.8 | 0.2 |
| LSC | *rps*3 | 657 | 657 | 657 | 657 | 0.00406 | 99.6 | 0.4 |
| LSC | *rpl*22 | 495 | 495 | 495 | 495 | 0.00404 | 99.6 | 0.4 |
| LSC | *rps*19 | 279 | 279 | 279 | 279 | 0 | 100 | 0 |
| **LSC TOTAL** | | **44076** | **44133** | **44136** | **44137** | **0.0031** | **99.6** | **0.4** |
| IR | *rpl*2 | 825 | 825 | 825 | 825 | 0.00647 | 99.4 | 0.6 |
| IR | *rpl*23 | 282 | 282 | 282 | 282 | 0 | 100 | 0 |
| IR | *ycf*2 | 6297 | 6297 | 6297 | 6297 | 0.00064 | 99.9 | 0.1 |
| IR | *ndh*B | 1533 | 1533 | 1533 | 1533 | 0 | 99.9 | 0.1 |
| IR | *rps*7 | 468 | 468 | 468 | 468 | 0 | 100 | 0 |
| IR | *rrn*16 | 1491 | 1491 | 1491 | 1491 | 0.00045 | 99.9 | 0.1 |
| IR | *rrn*23 | 2814 | 2814 | 2814 | 2814 | 0.00024 | 99.9 | 0.1 |
| IR | *rrn*4.5 | 103 | 103 | 103 | 103 | 0 | 100 | 0 |
| IR | *rrn*5 | 121 | 121 | 121 | 121 | 0 | 100 | 0 |
| **IR TOTAL** | | **13934** | **13934** | **13934** | **13934** | **0.0009** | **99.9** | **0.1** |
| SSC | *ndh*F | 2247 | 2253 | 2247 | 2253 | 0.00565 | 99.1 | 0.9 |
| SSC | *rpl*32 | 162 | 162 | 162 | 162 | 0.00412 | 99.6 | 0.4 |
| SSC | *ccs*A | 975 | 975 | 975 | 975 | 0.00479 | 99.5 | 0.5 |
| SSC | *ndh*D | 1530 | 1530 | 1530 | 1530 | 0.00610 | 99.4 | 0.6 |
| SSC | *psa*C | 246 | 246 | 246 | 246 | 0.00271 | 99.7 | 0.3 |
| SSC | *ndh*E | 306 | 306 | 306 | 306 | 0.00218 | 99.8 | 0.2 |
| SSC | *ndh*G | 531 | 531 | 531 | 531 | 0.00251 | 99.7 | 0.3 |
| SSC | *ndh*I | 504 | 504 | 504 | 504 | 0 | 100 | 0 |
| SSC | *ndh*A | 1092 | 1092 | 1092 | 1092 | 0.00367 | 99.6 | 0.4 |
| SSC | *ndh*H | 1182 | 1182 | 1182 | 1182 | 0.00620 | 99.4 | 0.6 |
| SSC | *rps*15 | 273 | 273 | 273 | 273 | 0.00977 | 99.0 | 1 |
| SSC | *ycf*1 | 5484 | 5493 | 5484 | 5496 | 0.00742 | 99.1 | 0.9 |
| **SSC TOTAL** | | **14532** | **14547** | **14532** | **14550** | **0.0046** | **99.5** | **0.5** |
| **TOTAL** | | **72542** | **72614** | **72602** | **72621** | **0.0029** | **99.7** | **0.3** |

LSC = large single copy; IR = inverted repeat; SSC = small single copy.

**Table S7**. Comparison of sequence length and nucleotide diversity in intergenic sequences of *Prangos fedtschenkoi* (*P.f*.), *Prangos lipskyi* (*P.l*.) and *Prangos trifida* (*P.t.*) plastomes

| Region | IGS | Size (bp) | | | Aligned  length  (bp) | Nucleotide diversity | Identity  (%) | Diversity  (%) |
| --- | --- | --- | --- | --- | --- | --- | --- | --- |
|  |  | (*P.f.*) | (*P.l*.) | (*P.t.)* |  |  |  |  |
| LSC | *trn*H-GUG/*psb*A | 192 | 192 | 194 | 193 | 0.01396 | 97.9 | 2.1 |
| LSC | *psb*A/*trn*K-UUU | 214 | 211 | 209 | 214 | 0.01276 | 97.2 | 2.8 |
| LSC | *trn*K-UUU/*mat*K | 229 | 229 | 229 | 229 | 0.00291 | 99.7 | 0.3 |
| LSC | *mat*K/*trn*K-UUU | 723 | 723 | 723 | 723 | 0.00553 | 99.4 | 0.6 |
| LSC | *trn*K-UUU/*rps*16 | 729 | 751 | 732 | 762 | 0.00833 | 95.4 | 4.6 |
| LSC | *rps*16/*trn*Q-UUG | 1185 | 1226 | 995 | 1263 | 0.03761 | 83.5 | 16.5 |
| LSC | *trn*Q-UUG/*psb*K | 351 | 351 | 351 | 351 | 0.00570 | 99.4 | 0.6 |
| LSC | *psb*K/*psb*I | 324 | 323 | 319 | 325 | 0.02103 | 96.3 | 3.7 |
| LSC | *psb*I/*trn*S-GCU | 140 | 140 | 140 | 140 | 0.00952 | 99.0 | 1 |
| LSC | *trn*S-GCU/*trn*G-GCC | 554 | 553 | 568 | 571 | 0.00847 | 96.8 | 3.2 |
| LSC | *trn*G-GCC/*trn*R-UCU | 152 | 159 | 155 | 165 | 0.00460 | 95.2 | 4.8 |
| LSC | *trn*R-UCU/*atp*A | 108 | 110 | 110 | 110 | 0 | 98.8 | 1.2 |
| LSC | *atp*A/*atp*F | 52 | 54 | 60 | 60 | 0 | 90.8 | 9.2 |
| LSC | *atp*F/*atp*H | 281 | 281 | 286 | 286 | 0.01423 | 97.4 | 2.6 |
| LSC | *atp*H/*atp*I | 773 | 765 | 784 | 793 | 0.00698 | 96.9 | 3.1 |
| LSC | *atp*I/*rps*2 | 265 | 273 | 273 | 281 | 0.00252 | 95.9 | 4.1 |
| LSC | *rps*2/*rpo*C2 | 236 | 233 | 237 | 237 | 0.01717 | 97.3 | 2.7 |
| LSC | *rpo*C2/*rpo*C1 | 211 | 211 | 211 | 211 | 0.00316 | 99.7 | 0.3 |
| LSC | *rpo*C1/*rpo*B | 5 | 5 | 5 | 5 | 0 | 100 | 0 |
| LSC | *rpo*B/*trn*C-GCA | 1236 | 1228 | 1222 | 1252 | 0.01160 | 96.5 | 3.5 |
| LSC | *trn*C-GCA/*pet*N | 684 | 679 | 697 | 693 | 0.00688 | 97.9 | 2.1 |
| LSC | *pet*N/*psb*M | 1116 | 1132 | 1132 | 1140 | 0.00717 | 97.6 | 2.4 |
| LSC | *psb*M/*trn*D-GUC | 688 | 689 | 688 | 689 | 0.00291 | 99.6 | 0.4 |
| LSC | *trn*D-GUC/*trn*Y-GUA | 121 | 121 | 121 | 121 | 0.01102 | 98.9 | 1.1 |
| LSC | *trn*Y-GUA/*trn*E-UUC | 76 | 76 | 76 | 76 | 0.01754 | 98.2 | 1.8 |
| LSC | *trn*E-UUC/*trn*T-GGU | 691 | 695 | 711 | 723 | 0.01808 | 94.5 | 5.5 |
| LSC | *trn*T-GGU/*psb*D | 1434 | 1443 | 1410 | 1450 | 0.00951 | 96.8 | 3.2 |
| LSC | *psb*D/*psb*C | - | - | - | - |  |  |  |
| LSC | *psb*C/*trn*S-UGA | 242 | 247 | 248 | 248 | 0.01377 | 97.6 | 2.4 |
| LSC | *trn*S-UGA/*psb*Z | 359 | 352 | 354 | 360 | 0.01136 | 97.4 | 2.6 |
| LSC | *psb*Z/*trn*G-GCC | 296 | 330 | 284 | 334 | 0.00685 | 88.6 | 11.4 |
| LSC | *trn*G-GCC/*trn*fM-CAU | 180 | 180 | 180 | 180 | 0.01111 | 98.9 | 1.1 |
| LSC | *trn*fM-CAU/*rps*14 | 164 | 164 | 163 | 164 | 0 | 98.8 | 1.2 |
| LSC | *rps*14/*psa*B | 123 | 114 | 123 | 123 | 0.00585 | 99.4 | 0.6 |
| LSC | *psa*B/*psa*A | 25 | 25 | 25 | 25 | 0 | 100 | 0 |
| LSC | *psa*A/*ycf*3 | 703 | 702 | 714 | 714 | 0.00475 | 98.4 | 1.6 |
| LSC | *ycf*3/*trn*S-GGA | 818 | 822 | 825 | 831 | 0.00735 | 98.1 | 1.9 |
| LSC | *trn*S-GGA/*rps*4 | 299 | 299 | 299 | 299 | 0.00446 | 99.6 | 0.4 |
| LSC | *rps*4/*trn*T-UGU | 366 | 385 | 372 | 386 | 0.01275 | 95.5 | 4.5 |
| LSC | *trn*T-UGU/*trn*L-UAA | 804 | 830 | 835 | 849 | 0.01205 | 95.0 | 5 |
| LSC | *trn*L-UAA/*trn*F-GAA | 363 | 370 | 363 | 370 | 0.00735 | 98.0 | 2 |
| LSC | *trn*F-GAA/*ndh*J | 355 | 355 | 377 | 377 | 0.00751 | 95.3 | 4.7 |
| LSC | *ndh*J/*ndh*K | 116 | 116 | 116 | 116 | 0.00575 | 99.4 | 0.6 |
| LSC | *ndh*K/*ndh*C | 48 | 46 | 46 | 48 | 0 | 97.2 | 2.8 |
| LSC | *ndh*C/*trn*V-UAC | 1115 | 1125 | 1143 | 1143 | 0.00839 | 97.3 | 2.7 |
| LSC | *trn*V-UAC/*trn*M-CAU | 175 | 175 | 174 | 175 | 0 | 100 | 0 |
| LSC | *trn*M-CAU/*atp*E | 190 | 187 | 187 | 190 | 0.00357 | 98.6 | 1.4 |
| LSC | *atp*E/*atp*B | - | - | - | - |  |  |  |
| LSC | *atp*B/*rbc*L | 760 | 759 | 761 | 761 | 0.00616 | 99.0 | 1 |
| LSC | *rbc*L/*acc*D | 602 | 602 | 603 | 604 | 0.00555 | 99.1 | 0.9 |
| LSC | *acc*D/*psa*I | 499 | 574 | 487 | 602 | 0.01780 | 83.5 | 16.5 |
| LSC | *psa*I/*ycf*4 | 397 | 402 | 397 | 402 | 0 | 99.2 | 0.8 |
| LSC | *ycf*4/*cem*A | 693 | 691 | 680 | 693 | 0 | 98.5 | 1.5 |
| LSC | *cem*A/*pet*A | 245 | 238 | 260 | 260 | 0.0084 | 93.5 | 6.5 |
| LSC | *pet*A/*psb*J | 635 | 661 | 951 | 977 | 0.00315 | 83.1 | 16.9 |
| LSC | *psb*J/*psb*L | 132 | 132 | 132 | 132 | 0 | 100 | 0 |
| LSC | *psb*L/*psb*F | 22 | 22 | 22 | 22 | 0 | 100 | 0 |
| LSC | *psb*F/*psb*E | 9 | 9 | 9 | 9 | 0 | 100 | 0 |
| LSC | *psb*E/*pet*L | 1010 | 1010 | 1010 | 1010 | 0.00924 | 99.1 | 0.9 |
| LSC | *pet*L/*pet*G | 152 | 152 | 152 | 152 | 0.00877 | 99.1 | 0.9 |
| LSC | *pet*G/*trn*W-CCA | 129 | 129 | 129 | 129 | 0 | 100 | 0 |
| LSC | *trn*W-CCA/*trn*P-UGG | 136 | 136 | 147 | 147 | 0.00490 | 94.4 | 5.6 |
| LSC | *trn*P-UGG/*psa*J | 386 | 386 | 378 | 387 | 0.00707 | 97.6 | 2.4 |
| LSC | *psa*J/*rpl*33 | 462 | 458 | 462 | 462 | 0.01019 | 98.4 | 1.6 |
| LSC | *rpl*33/*rps*18 | 185 | 185 | 161 | 185 | 0.01081 | 89.9 | 10.1 |
| LSC | *rps*18/*rpl*20 | 233 | 234 | 233 | 234 | 0.01149 | 98.3 | 1.7 |
| LSC | *rpl*20/*rps*12-2 | 770 | 769 | 766 | 770 | 0.00609 | 99.3 | 0.7 |
| LSC | *rps*12-2/*clp*P | 145 | 145 | 145 | 145 | 0.00460 | 99.5 | 0.5 |
| LSC | *clp*P/*psb*B | 451 | 449 | 449 | 451 | 0 | 99.6 | 0.4 |
| LSC | *psb*B/*psb*T | 210 | 200 | 195 | 210 | 0.01026 | 94.2 | 5.8 |
| LSC | *psb*T/*psb*N | 86 | 86 | 86 | 86 | 0.00775 | 99.2 | 0.8 |
| LSC | *psb*N/*psb*H | 102 | 102 | 102 | 102 | 0.00654 | 99.3 | 0.7 |
| LSC | *psb*H/*pet*B | 131 | 130 | 130 | 131 | 0.01538 | 98.0 | 2 |
| LSC | *pet*B/*pet*D | 172 | 179 | 179 | 179 | 0.00388 | 99.4 | 0.6 |
| LSC | *pet*D/*rpo*A | 184 | 133 | 131 | 138 | 0.00529 | 96.5 | 3.5 |
| LSC | *rpo*A/*rps*11 | 67 | 67 | 67 | 67 | 0.00995 | 99.0 | 1 |
| LSC | *rps*11/*rpl*36 | 113 | 113 | 113 | 113 | 0 | 100 | 0 |
| LSC | *rpl*36/*inf*A | 116 | 117 | 116 | 117 | 0.01724 | 97.7 | 2.3 |
| LSC | *inf*A/*rps*8 | 121 | 121 | 121 | 121 | 0 | 100 | 0 |
| LSC | *rps*8/*rpl*14 | 201 | 202 | 201 | 202 | 0.02985 | 96.7 | 3.3 |
| LSC | *rpl*14/*rpl*16 | 132 | 132 | 134 | 134 | 0.00505 | 98.5 | 1.5 |
| LSC | *rpl*16/*rps*3 | 153 | 147 | 153 | 153 | 0.00454 | 96.1 | 3.9 |
|  | *rps*3/*rpl*22 | - | - | - | - |  |  |  |
| LSC | *rpl*22/*rps*19 | 66 | 66 | 66 | 66 | 0 | 100 | 0 |
| **LSC TOTAL** | | **29418** | **29615** | **29594** | **30348** | **0.00756** | **97.2** | **2.8** |

**Table S7** (continued)

| Region | IGS | Size (bp) | | | Aligned length  (bp) | Nucleotide diversity | Identity  (%) | Diversity  (%) |
| --- | --- | --- | --- | --- | --- | --- | --- | --- |
|  |  | (*P.f.*) | (*P.l*.) | (*P.t.*) |  |  |  |  |
| IR | *rps*19/*rpl*2 | 58 | 54 | 59 | 59 | 0 | 93.2 | 6.8 |
| IR | *rpl*2/*rpl*23 | 18 | 18 | 18 | 18 | 0 | 100 | 0 |
| IR | *rpl*23/*trn*I-CAU | 165 | 165 | 165 | 165 | 0.00404 | 99.6 | 0.4 |
| IR | *trn*I-CAU/*ycf*2 | 88 | 88 | 88 | 88 | 0 | 100 | 0 |
| IR | *ycf*2/*trn*L-CAA | 586 | 586 | 590 | 590 | 0.00341 | 99.2 | 0.8 |
| IR | *trn*L-CAA/*ndh*B | 573 | 573 | 567 | 573 | 0.00118 | 99.2 | 0.8 |
| IR | *ndh*B/*rps*7 | 299 | 299 | 299 | 299 | 0.00223 | 99.8 | 0.2 |
| IR | *rps*7/*rps*12-2 | 55 | 55 | 55 | 55 | 0 | 100 | 0 |
| IR | *rps*12-2/  *trn*V-GAC | 1846 | 1847 | 1839 | 1849 | 0.00327 | 99.2 | 0.8 |
| IR | trnV-GAC/*rrn*16 | 227 | 227 | 227 | 227 | 0 | 100 | 0 |
| IR | *rrn*16/*trn*I-GAU | 294 | 294 | 294 | 294 | 0 | 100 | 0 |
| IR | *trn*I-GAU/  *trn*A-UGC | 64 | 64 | 64 | 64 | 0 | 100 | 0 |
| IR | *trn*A-UGC/*rrn*23 | 152 | 152 | 152 | 152 | 0 | 100 | 0 |
| IR | *rrn*23/*rrn*4.5 | 98 | 98 | 98 | 98 | 0 | 100 | 0 |
| IR | *rrn*4.5/*rrn*5 | 257 | 257 | 256 | 257 | 0 | 99.7 | 0.3 |
| IR | *rrn*5/*trn*R-ACG | 257 | 257 | 257 | 257 | 0.00519 | 99.5 | 0.5 |
| IR | *trn*R-ACG/*trn*N-GUU | 583 | 583 | 584 | 584 | 0.00573 | 99.2 | 0.8 |
| IR | *trn*N-GUU/*ycf*1 | 327 | 327 | 327 | 327 | 0.00204 | 99.8 | 0.2 |
| **IR TOTAL** | | **5947** | **5944** | **5939** | **5956** | **0.0015** | **99.4** | **0.6** |
| SSC | *ndh*F/*rpl*32 | 937 | 940 | 940 | 967 | 0.00727 | 95.5 | 4.5 |
| SSC | *rpl*32/*trn*L-UAG | 912 | 921 | 934 | 944 | 0.00514 | 96.9 | 3.1 |
| SSC | *trn*L-UAG/*ccs*A | 96 | 96 | 91 | 96 | 0.01465 | 95.1 | 4.9 |
| SSC | *ccs*A/*ndh*D | 223 | 223 | 223 | 223 | 0.01196 | 98.8 | 1.2 |
| SSC | *ndh*D/*psa*C | 88 | 88 | 89 | 89 | 0.01515 | 97.7 | 2.3 |
| SSC | *psa*C/*ndh*E | 266 | 266 | 266 | 266 | 0.00251 | 99.7 | 0.3 |
| SSC | *ndh*E/*ndh*G | 271 | 218 | 218 | 221 | 0.00459 | 98.2 | 1.8 |
| SSC | *ndh*G/*ndh*I | 361 | 361 | 361 | 361 | 0.00554 | 99.4 | 0.6 |
| SSC | *ndh*I/*ndh*A | 93 | 93 | 93 | 93 | 0.01434 | 98.6 | 1.4 |
| SSC | *ndh*A/*ndh*H | 1 | 1 | 1 | 1 | 0 | 100 | 0 |
| SSC | *ndh*H/*rps*15 | 91 | 91 | 91 | 91 | 0.00733 | 99.3 | 0.7 |
| SSC | *rps*15/*ycf*1 | 370 | 357 | 387 | 391 | 0.02083 | 92.1 | 7.9 |
| **SSC TOTAL** | | **3709** | **3655** | **3694** | **3743** | **0.0091** | **97.6** | **2.4** |
| **TOTAL** | | **39074** | **39214** | **39227** | **40047** | **0.0061** | **98.1** | **1.9** |

LSC = large single copy; IR = inverted repeat; SSC = small single copy; “-“ = no intergenic sequence between *psb*D and *psb*C genes.

**Table S8.** Comparison of sequence length and nucleotide diversity in introns of *Prangos fedtschenkoi* (*P.f*.), *Prangos lipskyi* (*P.l*.) and *Prangos trifida* (*P.t.*) plastomes

| Region | Intron | Size (bp) | | | Aligned length  (bp) | Nucleotide  diversity | Identity  (%) | Diversity  (%) |
| --- | --- | --- | --- | --- | --- | --- | --- | --- |
|  |  | (*P.f.*) | (*P.l*.) | (*P.t.*) |  |  |  |  |
| LSC | *rps*16 | 864 | 869 | 862 | 870 | 0.00465 | 98.8 | 1.2 |
| LSC | *trn*G-GCC | 710 | 709 | 698 | 700 | 0.00669 | 99.1 | 0.9 |
| LSC | *atp*F | 735 | 734 | 737 | 737 | 0.00455 | 99.3 | 0.7 |
| LSC | *rpo*C1 | 743 | 742 | 743 | 743 | 0.00629 | 99.3 | 0.7 |
| LSC | *ycf*3 | 788 | 793 | 783 | 798 | 0.00426 | 98.3 | 1.7 |
| LSC | *ycf*3 | 714 | 711 | 721 | 723 | 0.00940 | 97.9 | 2.1 |
| LSC | *trn*L-UAA | 520 | 493 | 506 | 525 | 0.00546 | 94.7 | 5.3 |
| LSC | *trn*V-UAC | 567 | 566 | 567 | 565 | 0.00355 | 99.5 | 0.5 |
| LSC | *clp*P | 641 | 638 | 637 | 647 | 0.01373 | 97.3 | 2.7 |
| LSC | *clp*P | 843 | 838 | 839 | 845 | 0.00797 | 98.5 | 1.5 |
| LSC | *pet*B | 750 | 750 | 756 | 756 | 0.01422 | 98.1 | 1.9 |
| LSC | *pet*D | 741 | 736 | 741 | 741 | 0.00362 | 99.2 | 0.8 |
| LSC | *rpl*16 | 948 | 927 | 949 | 956 | 0.01007 | 97.0 | 3 |
| **LSC total** | | **9564** | **9506** | **9539** | **9606** | **0.0073** | **98.23** | **1.77** |
| IR | *rpl*2 | 650 | 652 | 651 | 653 | 0.00411 | 99.3 | 0.7 |
| IR | *ndh*B | 682 | 682 | 682 | 682 | 0.00684 | 99.3 | 0.7 |
| IR | *rps*12-2 | 536 | 536 | 536 | 536 | 0 | 100 | 0 |
| IR | *trn*I-GAU | 944 | 944 | 950 | 946 | 0.00212 | 99.6 | 0.4 |
| IR | *trn*A-UGC | 812 | 814 | 815 | 815 | 0.00164 | 99.6 | 0.4 |
| **IR total** | | **3624** | **3628** | **3634** | **3632** | **0.0029** | **99.6** | **0.4** |
| SSC | *ndh*A | 1080 | 1078 | 1078 | 1080 | 0.0018 | 99.1 | 0.9 |
| **SSC total** | | **1080** | **1078** | **1078** | **1080** | **0.0018** | **99.1** | **0.9** |
| **TOTAL** | | **14268** | **14212** | **14251** | **14318** | **0.004** | **99.0** | **1.0** |

LSC = large single copy; IR = inverted repeat; SSC = small single copy.

**Table S9.** The shared presence of identical long tandem repeats (≥30 bp) of *Prangos* spp. in the plastomes of 25 accessions of Apiaceae and two of Araliaceae

| # | Species names | Family | GenBank  accession number | Tandem repeat number as designated in Table 7 for *Prangos fedtschenkoi* (*P.f.*)  and Table 8 for *P. lipskyi* (*P.l.*) | | | | | | | | | |
| --- | --- | --- | --- | --- | --- | --- | --- | --- | --- | --- | --- | --- | --- |
|  |  |  |  | #1 *P.f.* | #2 *P.f.*  #2 *P.l.* | #3 *P.f.* | #4 *P.f*  #3 *P.l.* | #5 *P.f.*  #5 *P.l.* | #6 *P.f.*  #6 *P.l.* | #7 *P.f.* | #1 *P.l.* | #4 *P.l.* | #7 *P.l.* |
|  | *Anethum graveolens* | Apiaceae | KR011055 | - | - | - | + | + | + | - | - | - | - |
|  | *Anethum graveolens* | Apiaceae | KR011055 | - | - | - | + | + | + | - | - | - | - |
|  | *Angelica acutiloba* | Apiaceae | KT963036 | - | - | - | - | + | - | - | - | - | - |
|  | *Angelica dahurica* | Apiaceae | NC029392 | - | - | - | - | + | - | - | - | - | - |
|  | *Angelica decursiva* | Apiaceae | KT781591 | - | - | - | - | + | - | - | - | - | - |
|  | *Angelica gigas* | Apiaceae | NC029393 | - | - | - | - | + | - | - | - | - | - |
|  | *Anthriscus cerefolium* | Apiaceae | GU456628 | - | - | - | + | + | - | - | - | - | - |
|  | *Bupleurum falcatum* | Apiaceae | KM207676 | - | - | - | - | - | + | - | - | - | - |
|  | *Bupleurum latissimum* | Apiaceae | NC033346 | - | - | - | - | - | - | - | - | - | - |
|  | *Carum carvi* | Apiaceae | NC029889 | - | - | - | + | - | - | - | - | - | - |
|  | *Coriandrum sativum* | Apiaceae | NC029850 | - | - | - | - | + | + | - | - | - | - |
|  | *Crithmum maritimum* | Apiaceae | HM596072 | - | + | - | + | + | + | - | - | - | - |
|  | *Daucus carota* | Apiaceae | DQ898156 | - | - | - | + | + | + | - | - | - | - |
|  | *Daucus carota* | Apiaceae | NC_008325.1 | - | - | - | + | + | + | - | - | - | - |
|  | *Foeniculum vulgare* | Apiaceae | KR011054 | - | - | - | + | + | + | - | - | - | - |
|  | *Glehnia littoralis* | Apiaceae | KT153022.1 | - | - | - | - | + | - | - | - | - | - |
|  | *Hansenia forbesii* | Apiaceae | NC035054 | - | - | - | + | + | + | - | - | - | - |
|  | *Hansenia forrestii* | Apiaceae | NC035056 | - | - | - | + | + | + | - | - | - | - |
|  | *Hansenia oviformis* | Apiaceae | NC035055 | - | - | - | + | + | + | - | - | - | - |
|  | *Hansenia weberbaueriana* | Apiaceae | NC035053 | - | - | - | + | + | - | - | - | - | - |
|  | *Ligusticum tenuissimum* | Apiaceae | KT963039.1 | - | - | - | - | + | + | - | - | - | - |
|  | *Ostericum grosseserratum* | Apiaceae | KT852844 | - | - | - | - | + | - | - | - | - | - |
|  | *Petroselinum crispum* | Apiaceae | HM596073 | - | - | - | + | + | - | - | - | - | - |
|  | *Prangos trifida* | Apiaceae | NC037852 | - | + | + | + | + | + | - | - | - | - |
|  | *Tiedemannia filiformis* | Apiaceae | HM596071 | - | - | - | + | + | + | - | - | - | - |
|  | *Hydrocotyle verticillata* | Araliaceae | HM596070 | - | - | - | - | + | - | - | - | - | - |
|  | *Fatsia japonica* | Araliaceae | KR021045 | - | - | - | - | + | - | - | - | - | - |

“+” = tandem repeats present; “-“ = tandem repeats absent.

**Table S10.** DNA sequences of the 5 bp inverted region (bold) and flanking inverted repeats in the *ndhB* intron of *Prangos* spp., 25 accessions of Apiaceae and two accessions of Araliaceae. The free energy of each secondary structure is indicated.

| # | Species names | Family | GenBank  accession number | Sequences | dG,  kcal/mol |
| --- | --- | --- | --- | --- | --- |
|  | *Prangos fedtschenkoi* | Apiaceae | KY652265 | ATCAAAAAAGAAAGAAGA**TGGGT**TCTTCTTTCTTTTTTGAT | -16.42 |
|  | *Prangos lipskyi* | Apiaceae | KY652266 | ATCAAAAAAGAAAGAAGA**ACCCA**TCTTCTTTCTTTTTTGAT | -16.72 |
|  | *Prangos trifida* | Apiaceae | NC037852 | ATCAAAAAAGAAAGAAGA**ACCCA**TCTTCTTTCTTTTTTGAT | -16.72 |
|  | *Anethum graveolens* | Apiaceae | KR011055 | ATCAAAAAAGAAAGAAGA**TGGGT**TCTTCTTTCTTTTTTGAT | -16.42 |
|  | *Anethum graveolens* | Apiaceae | NC029470 | ATCAAAAAAGAAAGAAGA**TGGGT**TCTTCTTTCTTTTTTGAT | -16.42 |
|  | *Angelica acutiloba* | Apiaceae | KT963036 | ATCAAAAAAGAAAGAAGA**TGAGT**TCTTCTTTCTTTTTTGAT | -16.42 |
|  | *Angelica dahurica* | Apiaceae | NC029392 | ATCAAAAAAGAAAGAAGA**TGAGT**TCTTCTTTCTTTTTTGAT | -16.42 |
|  | *Angelica decursiva* | Apiaceae | KT781591 | ATCAAAAAAGAAAGAAGA**TGAGT**TCTTCTTTCTTTTTTGAT | -16.42 |
|  | *Angelica gigas* | Apiaceae | NC029393 | ATCAAAAAAGAAAGAAGA**TGAGT**TCTTCTTTCTTTTTTGAT | -16.42 |
|  | *Anthriscus cerefolium* | Apiaceae | GU456628 | ATCAAAAAAGAAAGAAGA**ACTCA**TCTTCTTTCTTTTTTGAT | -16.72 |
|  | *Bupleurum falcatum* | Apiaceae | KM207676 | ATCAAAAAAGAAAGAAGA**TGGGT**TCTTCTTTCTTTTTTGAT | -16.42 |
|  | *Bupleurum latissimum* | Apiaceae | NC033346 | ATCAAAAAAGAAAGAAGA**TGGGT**TCTTCTTTCTTTTTTGAT | -16.42 |
|  | *Carum carvi* | Apiaceae | NC029889 | ATCAAAAAAGAAAGAAGA**ACTCA**TCTTCTTTCTTTTTTGAT | -16.72 |
|  | *Coriandrum sativum* | Apiaceae | NC029850 | ATCAAAAAAGAAAGAAGA**TGAGT**TCTTCTTTCTTTTTTGAT | -16.42 |
|  | *Crithmum maritimum* | Apiaceae | HM596072 | ATCAAAAAAGAAAGAAGA**TGAAT**TCTTCTTTCTTTTTTGAT | -16.42 |
|  | *Daucus carota* | Apiaceae | DQ898156 | ATCAAAAAAGAAAGAAGA**TGAGT**TCTTCTTTCTTTTTTGAT | -16.42 |
|  | *Daucus carota* | Apiaceae | NC_008325.1 | ATCAAAAAAGAAAGAAGA**TGAGT**TCTTCTTTCTTTTTTGAT | -16.42 |
|  | *Foeniculum vulgare* | Apiaceae | KR011054 | ATCAAAAAAGAAAGAAGA**TGGGT**TCTTCTTTCTTTTTTGAT | -16.42 |
|  | *Glehnia littoralis* | Apiaceae | KT153022.1 | ATCAAAAAAGAAAGAAGA**TGAGT**TCTTCTTTCTTTTTTGAT | -16.42 |
|  | *Hansenia forbesii* | Apiaceae | NC035054 | ATCAAAAAAGAAAGAAGA**TGAGT**TCTTCTTTCTTTTTTGAT | -16.42 |
|  | *Hansenia forrestii* | Apiaceae | NC035056 | ATCAAAAAAGAAAGAAGA**TGAGT**TCTTCTTTCTTTTTTGAT | -16.42 |
|  | *Hansenia oviformis* | Apiaceae | NC035055 | ATCAAAAAAGAAAGAAGA**TGAGT**TCTTCTTTCTTTTTTGAT | -16.42 |
|  | *Hansenia weberbaueriana* | Apiaceae | NC035053 | ATCAAAAAAGAAAGAAGA**TGAGT**TCTTCTTTCTTTTTTGAT | -16.42 |
|  | *Ligusticum tenuissimum* | Apiaceae | KT963039.1 | ATCAAAAAAGAAAGAAGA**TGAGT**TCTTCTTTCTTTTTTGAT | -16.42 |
|  | *Ostericum grosseserratum* | Apiaceae | KT852844 | ATCAAAAAAGAAAGAAGA**TGAGT**TCTTCTTTCTTTTTTGAT | -16.42 |
|  | *Petroselinum crispum* | Apiaceae | HM596073 | ATCAAAAAAGAAAGAAGA**TGGGT**TCTTCTTTCTTTTTTGAT | -16.42 |
|  | *Tiedemannia filiformis* | Apiaceae | HM596071 | ATCAAAAAATAAAGAAGA**TGAGT**TCTTCTTTCTTTTTTGAT | -16.42 |
|  | *Hydrocotyle verticillata* | Araliaceae | HM596070 | ATCAAAAAAGAAAGAAGA**ACTCA**TCTTCTTTCTTTT---AT | -12.08 |
|  | *Fatsia japonica* | Araliaceae | KR021045 | ATCAAAAAAGAAAGAAGA**ACTCA**TCTTCTTTCTTTT---AT | -12.08 |

**Table** **S11.** DNA sequences of the 9 bp inverted region (bold) and flanking inverted repeats in the *pet*B intron of *Prangos* spp., 25 accessions of Apiaceae and two accessions of Araliaceae. The free energy of each secondary structure is indicated.

| # | Species names | Family | GenBank  accession number | Sequences | dG,  kcal/mol |
| --- | --- | --- | --- | --- | --- |
|  | *Prangos fedtschenkoi* | Apiaceae | KY652265 | AAATCGAAA--GAAAGGTTTG**ACGACAAGA**-CAAACCTTTCTTTCGATTT- | -19.37 |
|  | *Prangos lipskyi* | Apiaceae | KY652266 | AAATCGAAA--GAAAGGTTTG**TCTTGTCGT**-CAAACCTTTCTTTCGATTT- | -19.98 |
|  | *Prangos trifida* | Apiaceae | NC037852 | AAATCGA--AAGAAAGGTTTG**TCTTGTCGT**-CAAACCTTTCTTTCGATTT- | - 18.79 |
|  | *Anethum graveolens* | Apiaceae | KR011055 | AAATCGAAA--GAAAGGTTTG**ACGACAAGA**-CAAACCTTTCTTTCGATTT- | -18.89 |
|  | *Anethum graveolens* | Apiaceae | NC029470 | AAATCGAAA--GAAAGGTTTG**ACGACAAGA**-CAAACCTTTCTTTCGATTT- | -18.89 |
|  | *Angelica acutiloba* | Apiaceae | KT963036 | AAATCGAAAAAGAAAGGTTTG**TCTTGTCGT**-CAAACCTTTCTTTCGATTT- | -14.79 |
|  | *Angelica dahurica* | Apiaceae | NC029392 | AAATCGAAAAAGAAAGGTTTG**TCTTGTCGT**-CAAACCTTTCTTTCGATTT- | -14.79 |
|  | *Angelica decursiva* | Apiaceae | KT781591 | AAATCGAAAAAGAAAGGTTTG**TCTTGTCGT**-CAAACCTTTCTTTCGATTT- | -14.79 |
|  | *Angelica gigas* | Apiaceae | NC029393 | AAATCGAAAAAGAAAGGTTTG**TCTTGTCGT**-CAAACCTTTCTTTCGATTT- | -14.79 |
|  | *Anthriscus cerefolium* | Apiaceae | GU456628 | AAATCGAAA--GAAAGGTTTG**TCTTTTCGT**-CAAACCTTTCTTTCGATTT- | -18.79 |
|  | *Bupleurum falcatum* | Apiaceae | KM207676 | AGATTAAGAAAATAAGTTTCA**AATTTTCTA**GTACATATCTGTTCCGACTA- | -4.34 |
|  | *Bupleurum latissimum* | Apiaceae | NC033346 | AGATTAAGAAAATAAGTTTCA**AATTTTCTA**GTACATATCTGTTCCGACTA- | -4.34 |
|  | *Carum carvi* | Apiaceae | NC029889 | AAATTGAAA--GAAAGGTTTG**ACGACAAGA**-CAAACCCTTCTTTCGATTT- | -11.60 |
|  | *Coriandrum sativum* | Apiaceae | NC029850 | AAATCGAAA--GAAAGGTTTG**TCTTGTCGT**-CAAACCTTTCTTTCGATTT- | -18.79 |
|  | *Crithmum maritimum* | Apiaceae | HM596072 | AAATCGAAA--GAAAGGTTTG**TCTTGTCGT**-CAAACCTTTCTTTCGATTT- | -18.79 |
|  | *Daucus carota* | Apiaceae | DQ898156 | AAATCGAAA--GAAAGGTTTG**TCTTTTCGT**-CAAGTCTTTCTTTCGATTT- | -12.57 |
|  | *Daucus carota* | Apiaceae | NC_008325.1 | AAATCGAAA--GAAAGGTTTG**TCTTTTCGT**-CAAGTCTTTCTTTCGATTT- | -12.57 |
|  | *Foeniculum vulgare* | Apiaceae | KR011054 | AAATCTAAA--GAAAGGTTTG**ACGACAAGA**-CAAACCTTTCTTTCGATTT- | -14.05 |
|  | *Glehnia littoralis* | Apiaceae | KT153022.1 | AAATCGAAAAAGAAAGGTTTG**TCTTGTCGT**-CAAACCTTTCTTTCGATTT- | -14.79 |
|  | *Hansenia forbesii* | Apiaceae | NC035054 | AAATCAAAA--GAAAGGTTTG**ACGAAAAGA**-CAAACCTTTCTTTCGATTT- | -13.34 |
|  | *Hansenia forrestii* | Apiaceae | NC035056 | AAATCAAAA--GAAAGGTTTG**ACGAAAAGA**-CAAACCTTTCTTTCGATTT- | -13.34 |
|  | *Hansenia oviformis* | Apiaceae | NC035055 | AAATCAAAA--GAAAGGTTTG**ACGAAAAGA**-CAAACCTTTCTTTCGATTT- | -13.34 |
|  | *Hansenia weberbaueriana* | Apiaceae | NC035053 | AAATCAAAA--GAAAGGTTTG**ACGAAAAGA**-CAAACCTTTCTTTCGATTT- | -13.34 |
|  | *Ligusticum tenuissimum* | Apiaceae | KT963039.1 | AAATCGAAA--GAAAGGTTTG**TCTTGTCGT**-CAAACCTTTCTTTCGATTT- | -18.79 |
|  | *Ostericum grosseserratum* | Apiaceae | KT852844 | AAATCGAAAAAGAAAGGTTTG**TCTTGTCGT**-CAAACCTTTCTTTCGATTT- | -14.79 |
|  | *Petroselinum crispum* | Apiaceae | HM596073 | AAATCGAAA--GAAAGGTTTG**TCTTGTCGT**-CAAACCTTTCTTTCGATTT- | -18.79 |
|  | *Tiedemannia filiformis* | Apiaceae | HM596071 | AAATCGAAA--GAAAGGTTTG**ACGAAAAGA**-CAAACCTTTCTGTCGATTT- | -16.01 |
|  | *Hydrocotyle verticillata* | Araliaceae | HM596070 | AAATCAAAA--GAAAGGTTTG**TCTTCTTTC**-AAATTCATTTTTATGCTTA- | -2.92 |
|  | *Fatsia japonica* | Araliaceae | KR021045 | -AAAAAATTTTCAAAGAATTA**T**--**AATTTG**-TAAATCGAAAGAAAGGTTTG | -3.00 |


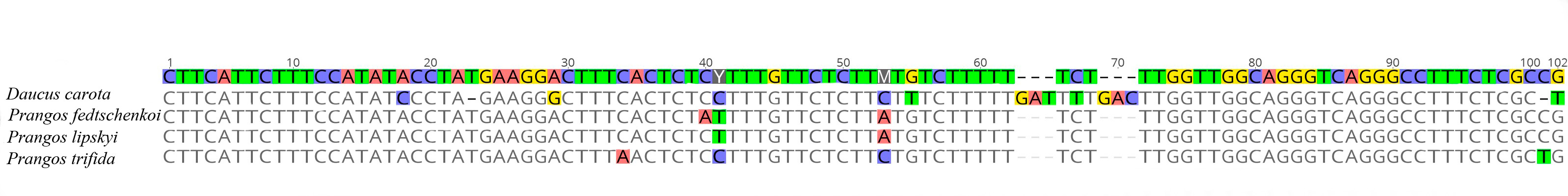


**Figure S1.** Extraction from MAUVE alignment of the novel fragments located in LSC region adjacent to the LSC/IRa within plastomes of *Prangos fedtschenkoi* (147 - 242 bp), *Prangos lipskyi* (501 - 596 bp), *Prangos trifida* (348 – 443 bp), and 101 bp fragment of mitochondrial IGS region adjacent to gene cytochrome b of *Daucus carota*. BLAST searches revealed a 100 bp match having 86% sequence identity of novel sequences of *Prangos fedtschenkoi* and *Prangos lipskyi*, and a 102 bp match and 88.2% of identity of the sequences from this region of *Prangos trifida* to noncoding mtDNA, specifically an intergenic spacer region adjacent to mitochondrial gene cytochrome b (cob) in three accessions of *Daucus carota* (as of 03 October 2018). Disagreements such as transitions and transversions are highlighted.
